# Low-Temperature Thermal Depolymerization of Polymethacrylates

**DOI:** 10.1021/jacs.6c13508

**Published:** 2026-08-29

**Authors:** Chih-Yin Lin, Nethmi De Alwis Watuthanthrige, Glen R. Jones, Stelios Andreou, Richard Whitfield, Hyun Suk Wang, Nghia P. Truong, Athina Anastasaki

**Affiliations:** Laboratory for Sustainable Polymers, Department of Materials, 111950 ETH Zürich, Vladimir-Prelog-Weg 5, Zürich 8093, Switzerland

## Abstract

The chemical recycling of poly­(methyl methacrylate) (PMMA) typically requires energy-intensive pyrolysis (>450 °C) or specialized photochemical setups restricted by light penetration depth. Herein, we report a purely thermal, low-temperature strategy that drives the efficient depolymerization of native, unaltered PMMA at 170 °C using commercially accessible peroxides. By maintaining a low steady-state radical concentration, oligomer formation can be fully eliminated, resulting in >95% of regenerated monomer under judiciously optimized conditions. Mechanistic studies and vapor-trapping experiments implicate a dual-activation pathway, where alkoxy radicals work alongside highly reactive chlorine radicals that undergo efficient hydrogen atom transfer with the polymer backbone, resulting in chain scission and subsequent depropagation to monomer. The full omission of chlorinated solvents could also be achieved, albeit at slightly higher temperatures. Notably, the real-world utility of this system is demonstrated by the direct depolymerization of commercial Plexiglas, despite the presence of comonomers, pigments, and additives.

## Graphical abstract

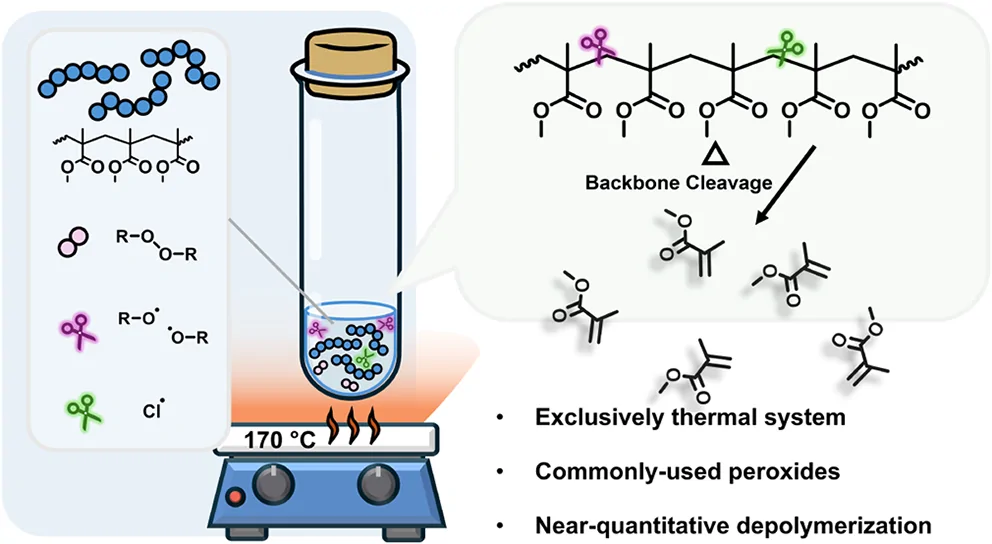

## Introduction

The accumulation of plastic waste represents one of the most pressing environmental challenges of the modern era, driving significant efforts toward a more circular plastic economy.^1^ Within this context, chemical recycling to monomer (depolymerization) has emerged as a promising solution for polymers which are thermodynamically susceptible to unzipping.^2^ An ideal candidate for this approach is poly­(methyl methacrylate) (PMMA), a widely utilized commodity polymer valued for its optical properties and frequently employed as a shatter-resistant alternative to glass.^3^ Thermodynamically, methyl methacrylate exhibits a relatively low ceiling temperature (*T*_c_ ∼ 295 °C in bulk). However, the corresponding polymer chains are inherently kinetically trapped, meaning that simply heating pristine PMMA above the *T*_c_ fails to achieve meaningful depolymerization due to the absence of active radical species required for depropagation to occur.^4–6^ Consequently, existing chemical recycling protocols rely on high-temperature pyrolysis, often using temperatures >450 °C to forcibly homolyze the robust carbon–carbon backbone and initiate radical generation.^7–10^ This conventional approach is energy-intensive and often triggers undesirable secondary side reactions that ultimately compromise the purity of the regenerated monomer.^11,12^

To alleviate these drawbacks, recent strategies have focused on lowering the requisite depolymerization temperature by exploiting alternative radical initiation pathways.^5,13^ For instance, significant progress has been achieved by leveraging the labile end-groups inherent to polymers synthesized via controlled radical polymerization techniques, such as atom transfer radical polymerization (ATRP) or reversible addition–fragmentation chain-transfer (RAFT) polymerization.^14^ These specialized terminal groups can be readily reactivated using external stimuli including light,^15–17^ radical initiators,^18,19^ or transition-metal catalysts,^20–22^ giving macroradicals capable of depropagation if above the ceiling temperature. Despite the success of this strategy, applying this approach to real plastic waste is unfeasible because the overwhelming majority of global PMMA production relies on conventional free radical polymerization and therefore lacks well-defined labile end-groups. To overcome this, the intentional introduction of weak linkages along the polymer backbone via copolymerization has also been explored. For example, Sumerlin and coworkers successfully integrated active ester methacrylate comonomers to facilitate midchain scission and depolymerization,^23^ while Diao and coworkers utilized small fractions of α-methylstyrene as weak linkages.^24^ In 2025, our group introduced a photothermal approach, whereby hydrogen atom transfer (HAT) chemistry was utilized to directly activate the inert backbone of PMMA, inducing chain scission and generating depropagating radicals without prior polymer modification. By employing the photoactivation of aromatic solvents such as 1,2,4-trichlorobenzene (TCB), high-yielding depolymerization of pristine PMMA was demonstrated significantly below conventional pyrolysis temperatures.^25^ Freakley and coworkers further validated these findings and expanded the scope to benzonitrile solvent with UV irradiation.^26^ Although these new methodologies enable the chemical recycling of unadulterated free radically synthesized polymers, they are fundamentally limited by the inherent constraints of photochemistry.^27^ Photochemical setups require specialized equipment and precise configurations, while the underlying physics remains highly dependent on light transmission.^28,29^ Consequently, limited photon penetration depth introduces severe bottlenecks when moving to larger reaction vessels. Considering that commercial pyrolysis operates exclusively under thermal conditions, a purely thermal methodology capable of driving the depolymerization of unaltered PMMA at significantly reduced temperatures while employing inexpensive, commercially available reagents would be highly desirable.

Herein, we demonstrate that common peroxides can efficiently initiate the thermal depolymerization of pristine PMMA at 170 °C in the complete absence of light or other additional external stimuli (Figure 1). Both batch and scheduled-dosing strategies have been explored to suppress the formation of oligomeric side products, without compromising the depolymerization yield, with the optimized approach achieving >95% monomer regeneration. Preliminary mechanistic investigations indicate a dual-activation pathway where initiator-derived alkoxy radicals directly attack the polymer backbone while simultaneously reacting with chlorinated solvents to liberate highly reactive chlorine radicals, which function as efficient HAT agents to enhance depolymerization. An alternative mechanism that fully omits the necessity for chlorine radicals was also investigated. Finally, we demonstrate the robustness of this thermal strategy by successfully depolymerizing a commercial sample of Plexiglas as well as by extending the scope to the other polymethacrylates.

**Figure 1:**
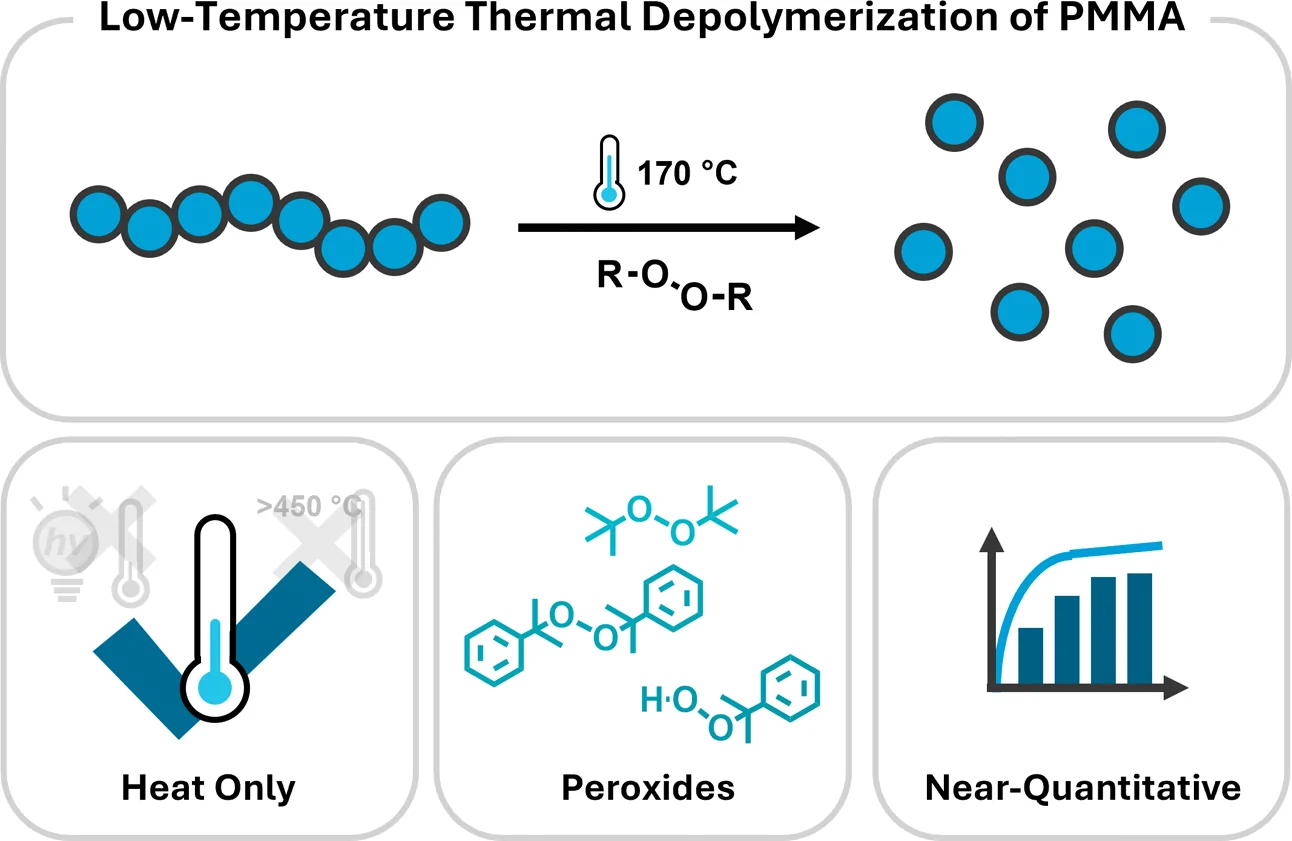
Low-temperature thermal chemical recycling of poly­(methyl methacrylate) (PMMA). Schematic representation of the peroxide-mediated thermal depolymerization of PMMA to methyl methacrylate (MMA) at 170 °C in the absence of light.

## Results and Discussion

In order to achieve low-temperature depolymerization of PMMA in the absence of light, we sought to identify a HAT agent that could be activated thermally. We focused our attention on peroxides, as they are inexpensive and commercially available in a wide variety of structural architectures. Furthermore, these reagents are well-established in organic synthesis for driving highly site-selective small-molecule HAT transformations,^30^ and they have been widely used for the radical-mediated grafting and modification of polymer backbones.^31–33^

We commenced our study by screening a series of peroxide architectures at 170 °C in TCB using PMMA synthesized by free radical polymerization (*M*_n_ = 337,000, *Đ* = 2.1; Figure S1) as a model polymer substrate, with an MMA repeat unit concentration (RUC) of 10 mM. A control experiment performed in the absence of a peroxide yielded negligible, if any, monomer regeneration after 23 h of reaction time, as determined by ^1^H NMR spectroscopy, confirming that the native polymer chains remain kinetically trapped under these milder thermal conditions (Figure 2a and Figure S2). Pleasingly, the addition of 1.0 equiv of benzoyl peroxide (BPO) relative to the polymer repeat unit successfully initiated depolymerization, though the monomer conversion was limited to 15%. This low efficiency is attributed to the short thermal half-life of BPO at 170 °C (Table S6), which results in rapid initial decomposition and premature radical termination.^34^ Furthermore, the initially generated benzoyloxy radicals could potentially undergo decarboxylation at this temperature,^35^ yielding carbon-centered phenyl radicals that are less effective for backbone HAT.^25^ To achieve a more sustained radical flux, we next investigated dicumyl peroxide (DCP). The higher thermal stability of DCP relative to BPO stems from its alkyl-substituted core, which lacks the electron-withdrawing carbonyl groups that weaken the peroxide bond in acyl architectures. Under identical reaction conditions, the use of 1.0 equiv of DCP yielded a significant increase in efficiency, achieving 56% monomer regeneration. We then evaluated di-*tert*-butyl peroxide (DTBP) as it exhibits greater thermal stability that might enable more sustained radical flux throughout the reaction. Satisfyingly, under identical conditions, the monomer conversion further increased to 66%, suggesting that these sterically unhindered *tert*-butoxyl radicals are well-suited for efficient backbone HAT reactions. Finally, we evaluated cumene hydroperoxide (CHP), the structurally related hydroperoxide to DCP. Because hydroperoxides thermally homolyze to generate both alkoxyl and highly reactive hydroxyl radicals simultaneously, we hypothesized that this system could enhance polymer backbone abstraction. Remarkably, utilizing 1.0 equiv of CHP under standard batch conditions gave the highest efficiency in this initial screen, affording 79% monomer conversion. The requirement for a full equivalent of initiator is attributed to the inherently low initiator efficiencies typical of peroxides at high temperatures in solution. Upon homolytic cleavage, solvent cage effects frequently cause rapid primary radical–radical recombination or secondary decay before the generated alkoxy radicals can diffuse into bulk solution and productively abstract hydrogen from the polymer backbone.^36^ Notably, size exclusion chromatography (SEC) analysis revealed significant decreases in molecular weight as the depolymerization progressed, where >80% reduction in *M*_n_ was achieved by the end of the reaction for all peroxide systems, indicating that chain scission occurred concurrently with depolymerization (Figure S3 and Table S7).

**Figure 2:**
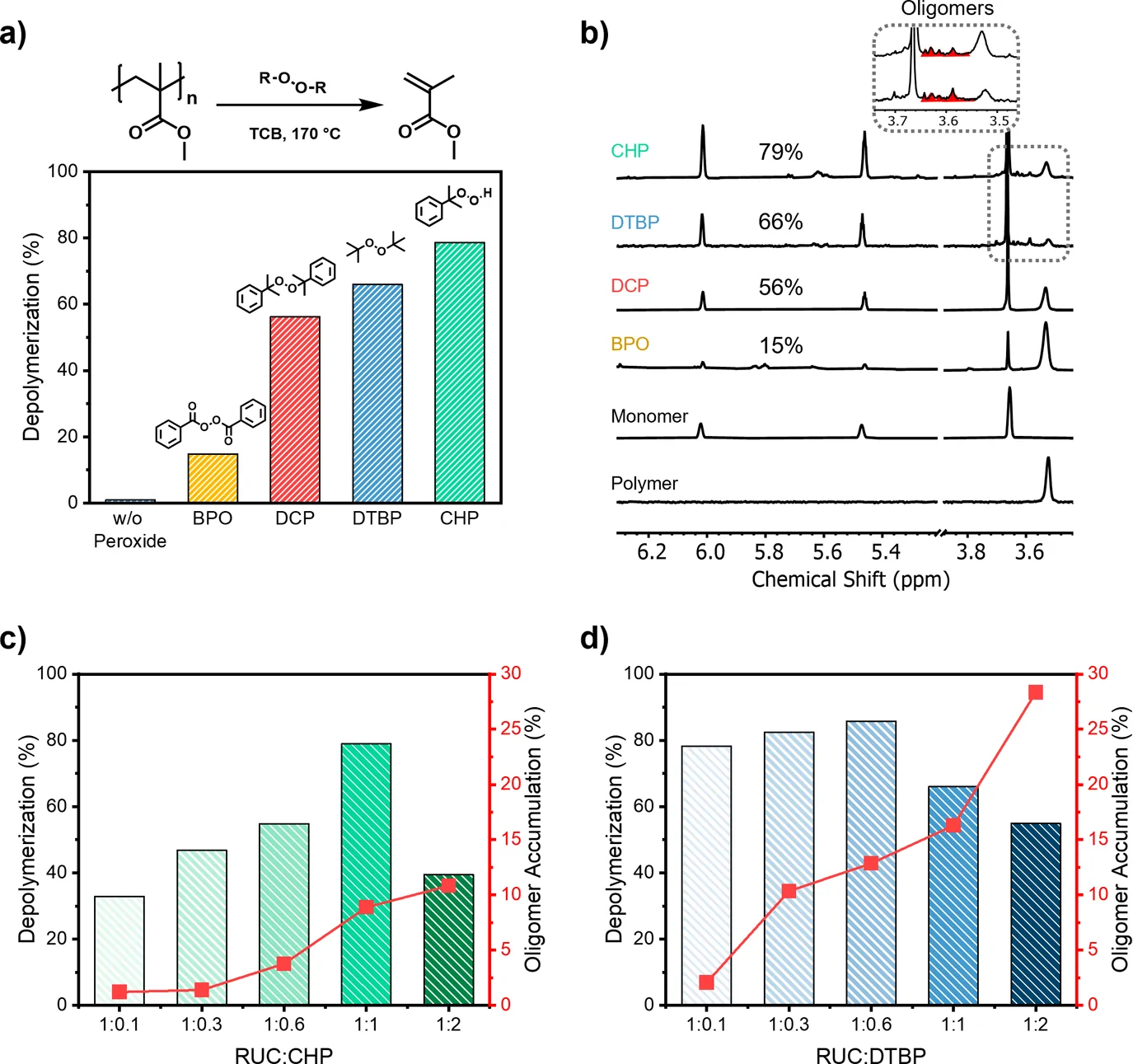
Peroxide initiator screening and loading optimization for PMMA depolymerization. (a) Monomer conversion of PMMA (*M*_n_ = 337,000, *Đ* = 2.1) in the presence of various peroxide architectures determined by ^1^H NMR spectroscopy (10 mM RUC, TCB, 170 °C, 23 h, [RU_0_]:[peroxide] = 1:1). (b) ^1^H NMR spectra of crude reaction mixtures showing characteristic vinyl proton signals of regenerated monomer and the presence of oligomeric side products. Evaluation of depolymerization conversion (bars, left axis) and oligomer accumulation (red lines, right axis) across varying initiator loadings for (c) cumene hydroperoxide (CHP) and (d) di-*tert*-butyl peroxide (DTBP).

We were highly encouraged by these initial screening results, which demonstrated that selecting a more thermally stable peroxide architecture can drive monomer regeneration up to 79% under purely thermal conditions. However, we sought to understand why the system plateaued at this stage and failed to achieve the quantitative conversions (>95%) observed in previously reported photochemical depolymerizations. To investigate this limitation, we analyzed the final reaction mixtures via ^1^H NMR spectroscopy (Figure 2b), which clearly displayed the distinctive vinyl proton signals of the generated MMA monomer at ∼5.5 and 6.0 ppm, alongside the sharp singlet at ∼3.7 ppm corresponding to the methyl ester protons of the monomer (Figure S4a). Crucially, however, a series of broad multiplets appeared in the region between the monomer and polymer signals (Figure S4b). These peaks are attributed to low-molecular-weight methyl methacrylate oligomers, suggesting the presence of a competing pathway that ultimately limits the monomer yield. Interestingly, while CHP performed better than DTBP, the residual polymer signal at ∼3.5 ppm in the DTBP reaction was instead weaker yet with more intense oligomer signals. This observation suggests that, in the case of DTBP, more polymer chains might be activated and consumed, but at the same time, more oligomer-forming side reactions were competing with depolymerization.

To investigate the formation of these oligomeric side products further, and to determine whether the depolymerization efficiency could be optimized, we selected our two most effective peroxides, CHP and DTBP, and conducted a series of reactions across a wide range of peroxide loadings. For CHP, lower loadings of peroxide (0.1, 0.3, and 0.6 equiv) were tested first (Figure 2c, Table S8). Depolymerization conversion dropped from 79% to 54% by decreasing the loading from 1.0 to 0.6 equiv, although the oligomer content was reduced to 4%. By further reducing the CHP loadings, 0.3 and 0.1 equiv showed 47% and 33% conversion respectively with lower oligomer amounts. Counterintuitively, increasing the CHP concentration to 2.0 equiv led to a lower depolymerization conversion of 40%, possibly due to the increased formation of oligomeric side products (11%). A different yet superior trend was observed for DTBP, where a 0.6 equiv loading afforded a markedly higher conversion of 86% with a lower level of oligomer formation (13%) compared to 1.0 equiv (Figure 2d and Table S9). Furthermore, while adjusting the DTBP loading to 0.3 and 0.1 equiv generated even less oligomers (10% and 2%, respectively), over 78% monomer could still be recovered under these conditions. Similar to the CHP system, when the DTBP loading was increased to 2.0 equiv, the depolymerization conversion dropped to 55% along with the significant rise in oligomers to 28%.

These observations clearly indicate that an excessive concentration of peroxide is detrimental to the reaction efficiency. This inhibition could be caused by high radical flux that accelerates bimolecular termination of unzipping macroradicals and concurrently drives a competitive reinitiation pathway where excess radicals polymerize the freshly generated monomer until hitting a thermodynamic ceiling (Figure S5). As established by Tobolsky and Szwarc in classic equilibrium studies, such chain-length-dependent thermodynamics can trap chain growth at the short oligomeric stage even above the ceiling temperature of polymer solution, effectively consuming liberated monomer.^37–39^

Consequently, we hypothesized that suppressing this competitive pathway required a shift away from batch protocols toward a metered addition strategy, thereby supplying a sufficient number of radicals while keeping the instantaneous radical concentration low enough to minimize oligomer formation. To test this, we monitored the depolymerization kinetics using DTBP, taking samples at regular intervals (Figure 3a, Figure S6 and Table S10). With an initial batch loading of 0.1 equiv, the reaction effectively ceased after approximately 3 h, ultimately yielding 78% monomer and 0.5% oligomer after 9 h (Figure 3b, Table S11). Increasing the batch loading to 0.3 equiv showed a similar profile, ceasing around the 3-h mark. While it afforded a slightly higher monomer yield of 83%, this came at the expense of significantly more oligomeric side products (9.4%) (Table S12). Pleasingly, when adopting a scheduled-dosing strategy and splitting 0.3 total equivalents into three separate aliquots, adding 0.1 equiv initially followed by subsequent 0.1 equiv additions at 3 and 6 h, the system achieved a final monomer conversion of 96% with just 0.5% oligomer (Table S13). This confirmed our hypothesis, proving that we can achieve almost quantitative conversions with minimal side products simply by spreading out the peroxide additions. This is likely due to the lower instantaneous radical concentration preventing oligomerization, while keeping the total amount of initiating radicals supplied to the system high.

**Figure 3:**
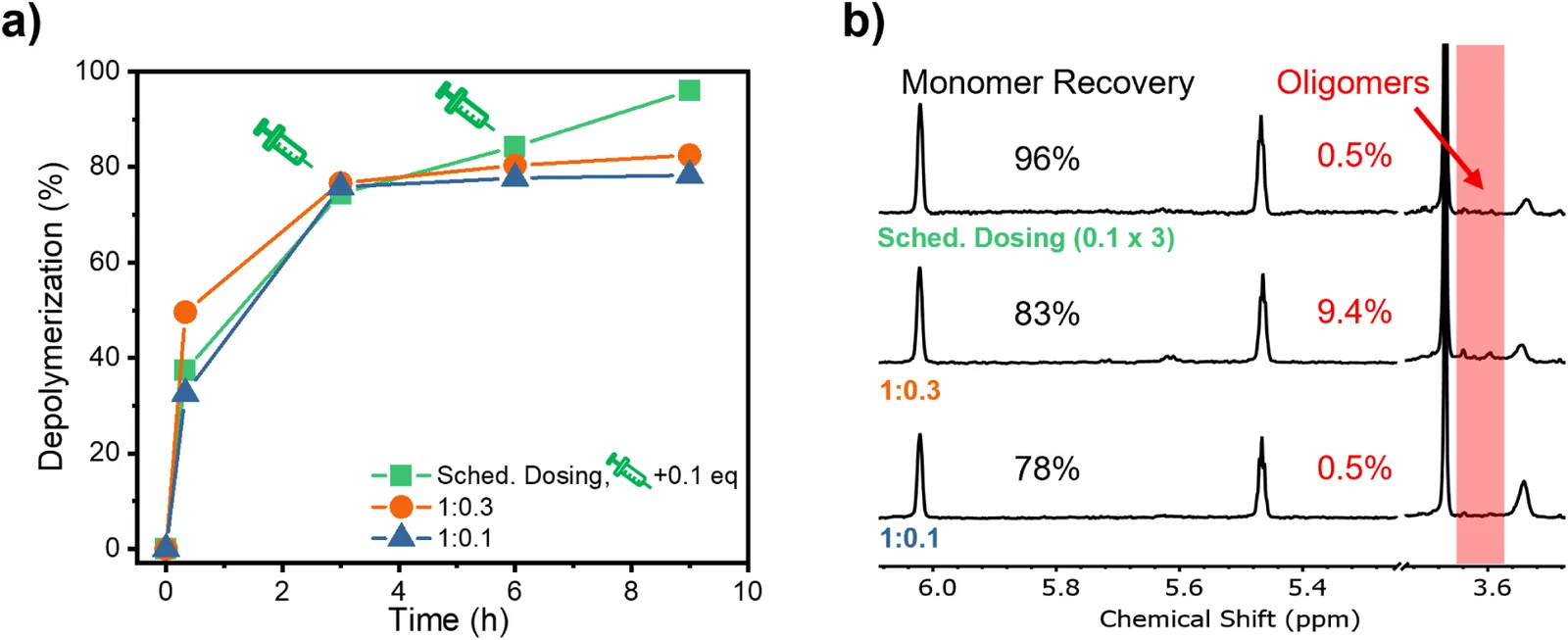
Reaction kinetics and optimization via a scheduled-dosing feeding strategy. (a) Kinetic profiles of DTBP-mediated PMMA depolymerization comparing single-batch additions (0.1 and 0.3 equiv) against a scheduled-dosing feeding protocol (3 × 0.1 equiv. added at 0, 3, and 6 h). Syringe icons denote injection points. (b) Corresponding final ^1^H NMR spectra highlighting the suppression of oligomeric side products under scheduled dosing conditions.

Having established that scheduled dosing enables >95% depolymerization while minimizing competing side reactions, we next sought to propose a clear mechanistic pathway. Although initially we considered a solely oxygen-centered radical mechanism due to the decomposition of peroxides, the necessity to use TCB puzzled us. Indeed, when switching to a nonchlorinated solvent, diphenyl ether (DPE), we envisioned equally high depolymerization efficiency, as it is high-boiling and lacks easily abstractable protons, making it a suitable candidate for HAT reactions at high temperatures (Figure S7). However, as seen in Figure 4a, while depolymerization using 0.6 equiv of DTBP at 170 °C did result in monomer formation, it yielded only 15% conversion (Table S14). Increasing the initiator loading to 1.2 or 3.0 equiv provided only marginal gains, resulting in 22% and 25% depolymerization, respectively (Table S14). We then investigated whether temperature was the limiting factor in this medium. By conducting the DTBP-mediated depolymerization at 190 and 220 °C, we obtained 18% and 45% depolymerization (Table S15). A similarly limited trend was observed for CHP, which gave 10%, 14%, and 51% depolymerization at 170 °C, 190 °C, and 220 °C, respectively (Table S16). Taken altogether, these results indicate a sole oxygen-centered pathway in the presence of DPE, albeit with lower depolymerization efficiency. Notably, these findings suggest that the presence of chlorine may be essential to achieve high percentages of regenerated monomer.

**Figure 4:**
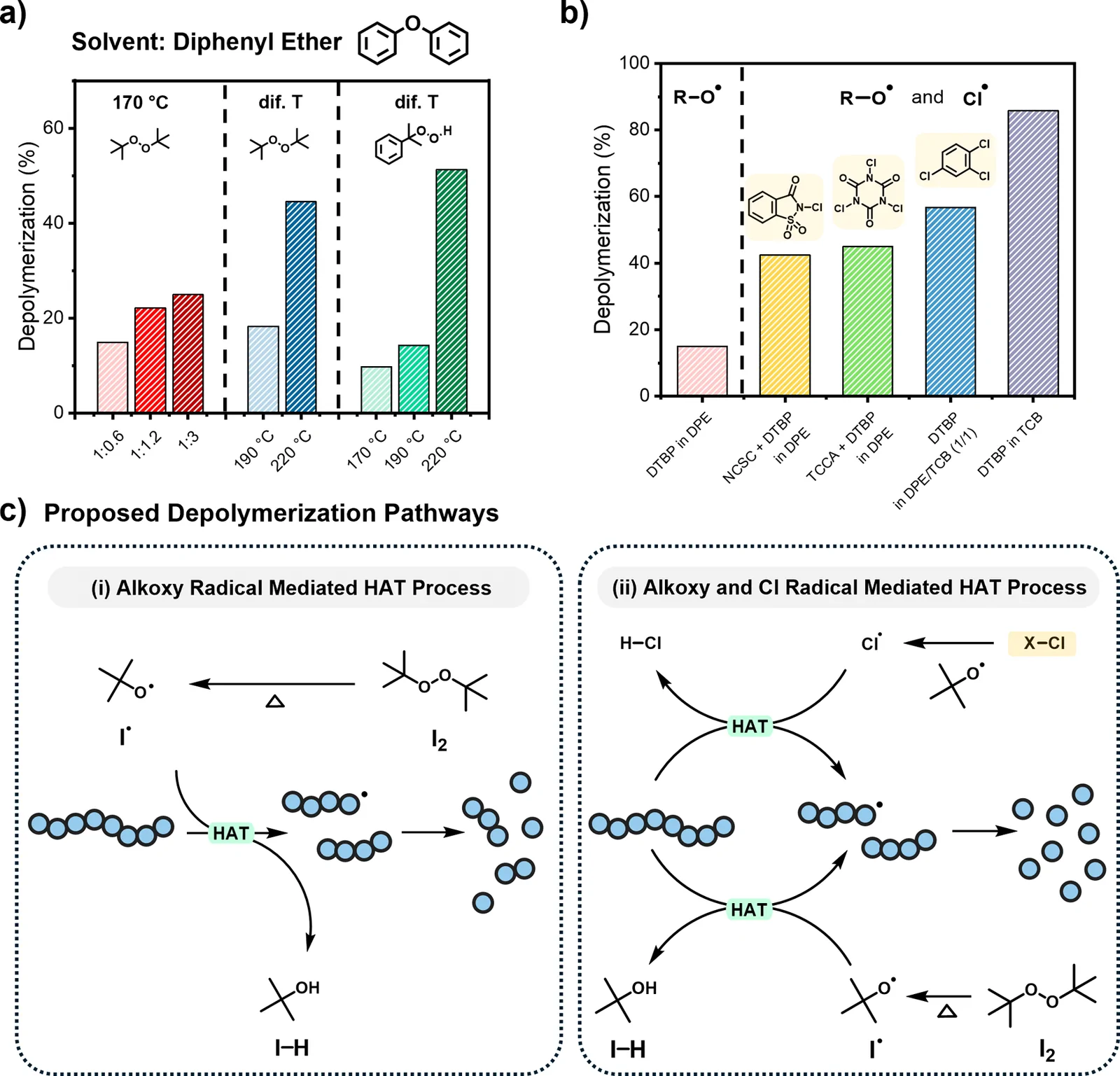
Investigation of solvent effects and proposed radical-mediated depolymerization pathways. (a) Depolymerization conversions in diphenyl ether (DPE) across varying initiator loadings and temperatures (170–220 °C). (b) Monomer conversion upon addition of external chlorine donors (N-chlorosaccharin (NCSC) or trichloroisocyanuric acid (TCCA)) or a 1:1 DPE/TCB solvent mixture. (c) Proposed mechanism showing (i) direct alkoxy radical-mediated hydrogen atom transfer (HAT) and (ii) parallel chlorine radical-mediated dual-activation.

Driven by these observations, we questioned whether the superior performance of our original system stemmed from direct solvent participation via chlorine radical intermediates (Figure 4b). We reasoned that if chlorine radicals were serving as highly active HAT mediators, introducing external chlorine sources into the poorly performing diphenyl ether medium should improve the depolymerization efficiency. To test this hypothesis, we spiked the DPE reactions with small quantities of chlorine donors. The addition of *N*-chlorosaccharin (NCSC) or trichloroisocyanuric acid (TCCA) alongside DTBP immediately triggered a dramatic improvement in monomer regeneration, increasing conversions from a baseline of 15% to 42% and 45%, respectively (Figure S8). Additionally, running the reaction in a 1:1 mixture of DPE and TCB further elevated the yield to 57% (Table S17). While these spiked systems do not fully replicate the 86% conversion achieved in pure TCB, the pronounced increase in monomer recovery provides compelling evidence that chlorine species actively accelerate depolymerization by opening a highly reactive, alternative HAT pathway.

To verify the generation of these transient halogen species, we sought direct evidence of hydrochloric acid (HCl) formation as an indication of chlorine radical activity. Vapor-trapping experiments during the heating of DTBP in TCB confirmed the evolution of HCl via rapid acidification of a methyl red indicator and the immediate formation of a silver chloride precipitate in a silver nitrate trap (see SI for full experimental details, Figure S9). Crucially, an identical HCl signature was observed when decomposing DTBP and TCCA in DPE whereas heating TCB in the absence of the peroxide yielded no acid evolution (Table S18). Further insight into this pathway was provided by GC-MS and NMR analysis of the TCB/DTBP postreaction mixture, which revealed the presence of dichloromethylbenzene (Figure S10). This finding highlights one of the complex competitive side reactions inherent to peroxide decay under these conditions, wherein thermal β-scission of the generated *tert*-butoxyl radical yields methyl radicals alongside acetone^36^ (Figure S11). These methyl radicals subsequently undergo homolytic aromatic substitution with the TCB solvent, generating the active chlorine radicals that abstract hydrogens from the polymer.^40,41^ These control experiments support the divergent pathways proposed in Figure 4c (ii). In nonchlorinated media, the alkoxy radicals likely abstract backbone hydrogen atoms directly from either methyl or methylene units of the PMMA backbone (Table S19); and the resulting midchain radical undergoes β-scission to yield a depropagating chain end (Figure S12, Figure 4c (i)). While this primary pathway also operates in chlorine-containing systems, the presence of chlorinated compounds introduces a highly efficient, parallel cycle facilitated by chlorine radicals. Because these chlorine radicals are highly reactive and exceptionally efficient HAT agents (Table S20),^25,42^ they significantly increase depolymerization, with the concurrent generation of HCl confirming their active participation (Figure 4c (ii)).

Finally, to demonstrate the applicability of this approach to real-world plastic waste, we applied our conditions to a commercial sample of colored Plexiglas. Under standard batch conditions at 170 °C with 1.0 equiv of DTBP, the system achieved 85% regeneration after 23 h (Figure S13), which improved to 90% upon implementing a scheduled dosing strategy. This performance demonstrates that the thermal methodology is fully viable for true commercial plastic samples, successfully tolerating the presence of colorants, minor comonomer fractions, and other undisclosed industrial additives without hindering the radical depropagation process. The optimized conditions could also be successfully applied to the depolymerization of other polymethacrylates including poly­(butyl methacrylate) (PBMA) and poly­(benzyl methacrylate) (PBzMA) (Figure S14).

## Conclusions

In conclusion, we have developed a purely thermal, low-temperature approach for the direct depolymerization of unadulterated poly­(methyl methacrylate) at 170 °C using commercially accessible peroxide initiators. By replacing conventional batch protocols with a scheduled-dosing strategy, we successfully suppressed competitive oligomerization pathways, driving monomer conversions to near-quantitative >95%. Mechanistic investigations and volatile-trapping experiments elucidated a dual-activation pathway wherein initiator-derived alkoxy radicals occur in parallel with a highly active chlorine radical HAT reaction. Crucially, the robustness of this system was validated by the efficient recycling of a commercial sample of Plexiglas. By removing the scalability and penetration depth bottlenecks inherent to photochemical methods, this purely thermal strategy provides a practical framework for the chemical recycling of pristine commodity plastics under accessible, lower-temperature conditions.

## Supplementary Material



## Notes

Experimental details, materials and methods, including photographs of experimental setups, tabulated data, ^1^H NMR spectra, and SEC chromatograms (PDF)
