# Supplementary material for: Low-Temperature Thermal Depolymerization of Polymethacrylates
Source: J Am Chem Soc. 2026 Aug 29;148(36):39220–6. doi: 10.1021/jacs.6c13508 (PMC13587657; doi:10.1021/jacs.6c13508)
Supplement: Supplementary file 1 [file ja6c13508_si_001.pdf]

Low-Temperature Thermal Depolymerization of Polymethacrylates

Chih-Yin Lin,[1] Nethmi De Alwis Watuthanthrige,[1] Glen R. Jones,[1] Stelios Andreou,[1] Richard Whitfield,[1] Hyun Suk Wang, [1] Nghia P. Truong,[1] Athina Anastasaki[1,*]

[1] Laboratory for Sustainable Polymers, Department of Materials, ETH Zürich, Vladimir-Prelog-Weg 5, 8093 Zürich, Switzerland

[*]Correspondence: athina.anastasaki@mat.ethz.ch

Table of Contents

1 Materials and Instrumentation **6**

1.1 Materials **6**

1.2 Instruments **6**

1.2.1 Size Exclusion Chromatography (SEC) **6**

1.2.2 ^{1}H Nuclear Magnetic Resonance **6**

1.2.3 Gas-Chromatography coupled with Mass Spectrometry (GC-MS) **7**

1.2.4 Setup of Depolymerization Reactor **7**

2 Experimental **8**

2.1 Synthesis Procedures **8**

2.1.1 Free Radical Polymerization (FRP) of PMMA **8**

2.2 Depolymerization Procedures **8**

2.2.1 Depolymerization of PMMA Synthesized via FRP for Peroxides Screening **8**

2.2.1.1 Depolymerization of PMMA with Benzyl Peroxide (BPO) **8**

2.2.1.2 Depolymerization of PMMA with Dicumyl Peroxide (DCP) **9**

2.2.1.3 Depolymerization of PMMA with Di-*tert*-butyl Peroxide (DTBP) **9**

2.2.1.4 Depolymerization of PMMA with Cumyl Hydroperoxide (CHP) **9**

2.2.1.5 Control Experiment: Depolymerization of PMMA without Peroxides **9**

2.2.2 Peroxide Loading in Depolymerization of FRP-PMMA **10**

2.2.2.1 Depolymerization using Different Loadings of CHP **10**

2.2.2.2 Depolymerization using Different Loadings of DTBP **11**

2.2.3 Depolymerization of FRP-PMMA via Scheduled Dosing Methodology **11**

2.2.3.1 Depolymerization with Scheduled Dosing DTBP **11**

2.2.3.2 Kinetic Experiment **12**

2.2.4 Depolymerization of the FRP-PMMA in Diphenyl Ether **13**

2.2.4.1 Depolymerization with DTBP in Diphenyl Ether **13**

2.2.4.2 Depolymerization with Increasing Temperature with DTBP in Diphenyl Ether 14

2.2.4.3 Depolymerization with Increasing Temperature with CHP in Diphenyl Ether 14

2.2.4.4 Depolymerization Using N-chlorosaccharin (NCSC) as Chlorine Source in Diphenyl Ether 14

2.2.4.5 Depolymerization Using Trichloroisocyanuric acid (TCCA) as Chlorine Source in Diphenyl Ether 15

2.2.4.6 Depolymerization of PMMA with DTBP Using Co-solvent, TCB/DPE 15

2.2.5 Depolymerization of PMMA Synthesized via FRP with Azo-Initiator 15

2.2.5.1 Depolymerization of the PMMA with 1,1′-Azobis(cyclohexanecarbonitrile) (ABCN) in Trichlorobenzene (TCB) 15
2.2.5.2 Depolymerization Using N-chlorosaccharin (NCSC) as Chlorine Source in Diphenyl Ether 16
2.2.5.3 Depolymerization Using Trichloroisocyanuric acid (TCCA) as Chlorine Source in Diphenyl Ether 16
2.2.6 Depolymerization of the Commercial PMMA 17
2.2.6.1 Depolymerization of the Commercial PMMA with Batch Condition 17
2.2.6.2 Depolymerization of the Commercial PMMA with Scheduled Dosing Condition 17
2.2.7 Depolymerization of the Other Polymethacrylates 18
2.2.7.1 Depolymerization of Poly(Benzyl Methacrylate) (PBzMA) 18
2.2.7.2 Depolymerization of Poly(n-Butyl Methacrylate) (PBMA) 19
2.3 Vapor-Trapping Experiments for Confirmation of Hydrochloric acid (HCl) formation . 20
2.3.1 HCl Formation Confirmation under Condition of DTBP in TCB Using Acid Formation and Chloride Salt Formation Indicator 20
2.3.2 HCl Formation Confirmation under Condition of PMMA and DTBP in TCB Using Acid Formation and Chloride Salt Formation Indicator 20
2.3.3 HCl Formation Confirmation under Condition of DTBP and TCCA in DPE Using Acid Formation and Chloride Salt Formation Indicator 21
2.3.4 HCl Formation Confirmation under Condition of TCB alone Using Acid Formation and Chloride Salt Formation Indicator 21
2.4 MMA Oligomerization 21
2.4.1 MMA Oligomerization with Standard Batch Condition of DTBP 21
3 Additional Data **23**
3.1 Characterization of PMMA Synthesized via FRP 23
3.2 Control Experiments – Depolymerization without Peroxide 23
3.3 Peroxide Screening for Depolymerization of PMMA 24
3.4 Optimization of Peroxides Loadings 27
3.5 Kinetic Profile of the Depolymerization with or without Scheduled Dosing Strategy 29
3.6 Additional Choice of Solvents for HAT Reaction-Triggered Depolymerization 31
3.7 Depolymerization of PMMA in non-Chlorinated Solvent, Diphenyl Ether (DPE) 31
3.8 Proposed Mechanism of Mid-Chain Depolymerization of PMMA via Dual-Activation Pathway 34
3.9 Radical Electrophilicity and Bond Dissociation Energies 37
3.10 Depolymerization of Commercial PMMAs 40
3.11 Depolymerization of Other Polymethacrylates 41

List of Figures

Figure S1 Characterization of PMMA synthesized via FRP: a) SEC trace (M_n = 337,000, Đ = 2.1), b) ^{1}H NMR spectrum after purification. 23

Figure S2 Control experiment with trichlorobenzene (TCB). RUC of 10 mM PMMA in TCB was reacted for 23 h at 170 °C a) SEC traces of t_{0h} and t_{23h}. b) ^{1}H NMR showed a negligible amount of monomer recovery. 23

Figure S3 SEC traces of PMMA depolymerization using various peroxides. Reaction conditions: PMMA (10 mM RUC) and peroxide (1.0 eq.) in TCB at 170 °C for 23 h, showing concurrent chain scission and depolymerization. 25

Figure S4 Example of determination of a) depolymerization conversion by NMR analysis, where in this case, depolymerization conversion is 82.35%; and b) oligomer amount by NMR analysis, where in this case, oligomer amount is 8.8%. 26

Figure S5 Thermodynamic feasibility of oligomer formation with 10 mM MMA. a) Reaction schemes MMA polymerization vs. PMMA depolymerization under identical conditions ($[RUC]_0$ or $[MMA]_0$: DTBP = 1 : 0.6, 170 °C, 23 h); b) evolution of MMA and oligomer content; c) ^{1}H NMR product analysis demonstrating oligomer formation with 10 mM MMA, without high-molecular-weight polymer formation. 28

Figure S6 Evolution of molecular weight distributions during the DTBP-mediated depolymerization of PMMA, as monitored by SEC. RUC of 10 mM PMMA and 0.6 eq DTBP in trichlorobenzene was reacted for 9 h at 170 °C, as significant concurrent chain scission and depolymerization happened in the reaction. 29

Figure S7 Venn diagram for solvent selection 31

Figure S8 Control experiments evaluating PMMA depolymerization with external chlorine sources. **a)** Depolymerization using DTBP alone, TCCA alone, and a combination of DTBP and TCCA. **b)** Depolymerization using DTBP alone, NCSC alone, and a combination of DTBP and NCSC. **c)** Comparison of different thermal initiators (DTBP vs. ABCN) for PMMA depolymerization (Reaction conditions: RUC of 10 mM PMMA, 0.6 eq DTBP or 1 eq ABCN, and 0.1 eq chlorine donor at 170 °C in DPE). 33

Figure S9 a) Experimental setup for vapor trapping used to confirm HCl generation. b) Compositions of indicator solutions for acid detection (yellow at basic pH and red at acidic pH) and chloride detection (AgCl precipitation in the presence of Cl^- ions). 34

Figure S10 a) GC-MS of the major nonvolatile byproduct formed after heating DTBP in TCB at 170 °C for 15 h. b) ^{1}H NMR of the major nonvolatile byproduct formed after heating DTBP in TCB at 170 °C for 15 h. c) ^{13}C NMR of the major nonvolatile byproduct formed after heating DTBP in TCB at 170 °C for 15 h. 36

Figure S11 Possible pathway of the formation of dichloromethylbenzene during the reaction of DTBP in TCB. 37

Figure S12 Proposed mechanism for mid-chain scission and depolymerization via alkoxy radical and chlorine radical. 39

Figure S13 a) Photograph of blue Plexiglas pieces used in the demonstration of commercial PMMA depolymerization. b) Depolymerization of blue Plexiglas under various reaction conditions, as determined by ^{1}H NMR spectroscopy.. 40
Figure S14 a) Depolymerization of poly(benzyl methacrylate) using DTBP in TCB, and b) depolymerization of poly(butyl methacrylate) using DTBP in TCB, determined via ^{1}H NMR analysis.. 41

List of Tables

Table S1 Reaction solution preparation for optimization of CHP loading.................................. 10
Table S2 Reaction solution preparation for optimization of DTBP loading 11
Table S3 Reaction solution preparation for different DTBP loadings in diphenyl ether 13
Table S4 Scheduled dosing stock solution preparation for depolymerization of commercial PMMA 18
Table S5 Reaction solution preparation for depolymerization of commercial PMMA 18
Table S6 1 h half-life temperatures for peroxides. 24
Table S7 Depolymerization conversions determined by NMR and SEC analysis§ 24
Table S8 Evolution of depolymerization conversion and oligomer percentage determined by NMR analysis for the depolymerization of PMMA using X eq (where X = 0.1-2 eq) CHP § 27
Table S9 Evolution of depolymerization conversion and oligomer percentage determined by NMR analysis for the depolymerization of PMMA using X eq (where X = 0.1-2 eq) DTBP § .. 27
Table S10 Depolymerization conversion determined by NMR and SEC, and M_n and M_n shift compared to the initial polymer.§ 29
Table S11 Depolymerization conversions and oligomer percentage determined by NMR analysis for the depolymerization of PMMA using 0.1 eq DTBP.§ 30
Table S12 Depolymerization conversions and oligomer percentage determined by NMR analysis for the depolymerization of PMMA using 0.3 eq DTBP.§ 30
Table S13 Depolymerization conversions and oligomer percentage determined by NMR analysis for the depolymerization of PMMA using 0.3 eq DTBP with scheduled dosing strategy (initially 0.1 eq and subsequent 0.1 eq additions at 3 and 6 hrs).§.. 30
Table S14 Depolymerization conversions determined by NMR analysis for the depolymerization of PMMA using varying equivalents of DTBP in DPE at 170 °C.§.. 31
Table S15 Depolymerization conversions determined by NMR analysis for the depolymerization of PMMA using DTBP in DPE at different temperatures.§ 32
Table S16 Depolymerization conversions determined by NMR analysis for the depolymerization of PMMA using CHP in DPE at different temperatures.§... 32
Table S17 Depolymerization conversions determined by NMR analysis for the depolymerization of PMMA using DTBP in DPE with external chlorine sources.. 32
Table S18 Hydrochloric acid formation confirmed by vapor-trapping experiments. 35

Table S19 Electrophilicity of chlorine radical and alkoxyl radical or hydroxyl radical derived from peroxide species.[3] 37
Table S20 Comparison of C–H bond dissociation energies (BDE) across diverse chemical environments. [4] 38

1 Materials and Instrumentation

1.1 Materials

All materials were obtained from Sigma Aldrich, Fischer Scientific, or TCI and used as received, while those which were pretreated or from other sources are stated below separately. Methyl methacrylate was purified via passing through basic alumina column before use. *N*-Chlorosaccharin was obtained from Fluorochem Ltd, while trichloroisocyanuric acid was obtained from Sigma Aldrich. Poly(methyl methacrylate) (PMMA) standards for calibration of SEC were purchased from Aglient Technologies (PMMA EasiVials). PLEXIGLAS® sheets were purchased from POLYVANTIS.

1.2 Instruments

1.2.1 Size Exclusion Chromatography (SEC)

Size exclusion chromatography was performed on a Shimadzu instrument, equipping with a SIL-20A HT automatic sampler, LC-20AD pump liquid chromatogram, CBM-20A communication module, and CTO-20A column oven to maintain SEC measurement temperature at 40 °C (Columns: 50 x 7.5 mm guard column with 10 µm bead size, and three 300 x 8 mm KF-805L columns with 10 µm bead size, which pore size maximum is 5000 Å). SPD-20A detector and RID-20A detector were used for UV/VIS and refractive index recording respectively. N, N-dimethylacetamide (DMAc, Acros, HPLC grade, with 0.03 w/v LiBr) was used as eluent and the flow rate was set to 1 mL/min. Poly(methyl methacrylate) standards were used for calibration with the molecular weight range from 7,000 to 1,600,000 g/mol. All SEC samples were dissolved in DMAc and filtered by 0.45 µm PTFE syringe prior to analysis.

1.2.2 ^{1}H Nuclear Magnetic Resonance

A Bruker Avance-400 MHz spectrometer with a cryo-probe was used for NMR analysis. $CDCl_3$ or acetone-d_6 were used as NMR solvents. Chemical shifts are shown in ppm downfield from tetramethylsilane (TMS) and referenced to residual solvent proton signals.

1.2.3 Gas-Chromatography coupled with Mass Spectrometry (GC-MS)

Thermo Scientific QExative GC Orbitrap with Ion-Trap (Orbitrap) and electron impact (EI) as the ion source was used. Mass spectrometry experiments were operated on a Bruker Maxis I quadrupole-time-of-flight (QTOF) at 4500V emitter voltage with electrospray ionization (ESI) as ion source.

To prepare the sample, 2 mL reaction solution was dried with air blowing in a 5 mL vial to remove the residual solvent (TCB) and isolate the solid byproducts. The solid residue was then transferred to the sampling tube for GC-MS analysis.

1.2.4 Setup of Depolymerization Reactor

A Schlenk tube equipped with a vacuum valve with PTFE screwed stopcock plug was used (purchased from FengTecEx GmbH) to prevent monomer leakage during the reaction.

The side vacuum line was sealed with a rubber septum for ease of degassing. The reactor tube was placed in the oil bath in a beaker, and the internal liquid level was approximately 1 cm below the oil surface as illustrated in **Scheme S1**.

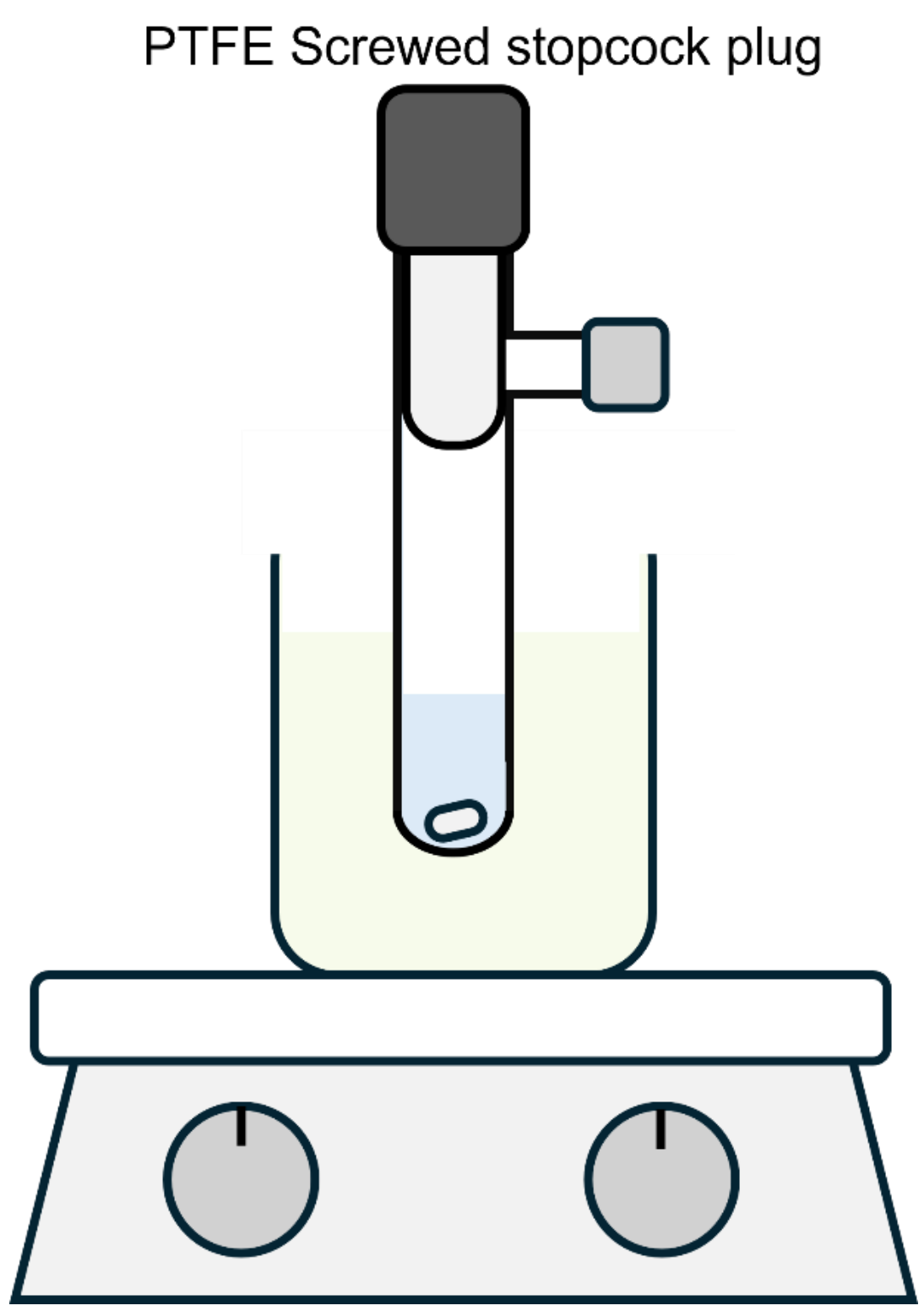

Scheme S1 Depolymerization Reactor Setup

2 Experimental

2.1 Synthesis Procedures

2.1.1 Free Radical Polymerization (FRP) of PMMA

A volume of 12 mL methyl methacrylate (MMA, 10.2 g, 101.9 mmol) and 3.8 mg 2,2'-azobis(isobutyronitrile) (AIBN, 23.4 μmol) were added into 24 mL acetonitrile in a 50 mL round bottom flask, a stir bar was put in the flask before being sealed with a septum. The solution was deoxygenated by nitrogen purging for 15 minutes, and the polymerization was conducted in an oil bath at 70 °C for 15 hours (stirring rate = 400 rpm). The flask was removed from the oil bath and cooled to room temperature. The polymerization was stopped by removing the septum and exposing the reaction mixture to air. The purification was done by precipitating in methanol three times, and the polymer (FRP-PMMA) was dried in a vacuum overnight and checked by ^{1}H NMR to ensure there was no monomer residue.

2.2 Depolymerization Procedures

2.2.1 Depolymerization of PMMA Synthesized via FRP for Peroxides Screening

2.2.1.1 Depolymerization of PMMA with Benzyl Peroxide (BPO)

Stock solutions were prepared as described as follows: 20 mg of PMMA was dissolved in 10 mL trichlorobenzene, and 24.2 mg BPO was dissolved in 5 mL trichlorobenzene.

1 mL PMMA stock solution and 1 mL BPO stock solution were mixed ($[RU_0]$ = 10 mM, RU_0 : BPO = 1 : 1) in a 20 mL Schlenk glass tube with a magnetic stir bar. The tube was sealed with a PTFE stopcock plug at the top and a rubber septum at the side. The solution was deoxygenated via nitrogen purging for 15 minutes. The vessel was then submerged in a 170 °C oil bath (stirring at 400 rpm), waith the internal liquid level approximately 1 cm below the oil surface. The reaction was quenched after 23 h by removing the vessel from the heat and cooling to room temperature.

A 0.35 mL aliquot was taken for ^{1}H NMR and SEC analyses. To prepare the SEC samples, 0.3 mL solution was transferred to a 5 mL vial, dried under a stream of air, and redissolved in 0.35 mL DMAc. The resulting solution was filtered through a 0.45 μm PTFE membrane

syringe filter into a vial equipped with an insert. For ^{1}H NMR analysis, the remaining 50 μL aliquot was diluted in $CDCl_3$.

2.2.1.2 Depolymerization of PMMA with Dicumyl Peroxide (DCP)

The depolymerization of PMMA using DCP was conducted following the procedure established for BPO (section 2.2.1.1) with stock solutions prepared as follows:

20 mg of PMMA was dissolved in 10 mL of trichlorobenzene, and 27 mg DCP was dissolved in 5 mL of trichlorobenzene. 1 mL PMMA stock solution and 1 mL DCP stock solution were mixed in a 20 mL Schlenk glass tube to reach the initial concentration ($[RU_0]$ = 10 mM, RU_0 : DCP = 1 : 1).

2.2.1.3 Depolymerization of PMMA with Di-*tert*-butyl Peroxide (DTBP)

The depolymerization of PMMA using DTBP was conducted following the procedure established for BPO (section 2.2.1.1) with stock solutions prepared as follows:

20 mg of PMMA was dissolved in 10 mL of trichlorobenzene, and 18 μL DTBP was added in 5 mL of trichlorobenzene. 1 mL PMMA stock solution and 1 mL DTBP stock solution were mixed in a 20 mL Schlenk glass tube to reach the initial concentration ($[RU_0]$ = 10 mM, RU_0 : DTBP = 1 : 1).

2.2.1.4 Depolymerization of PMMA with Cumyl Hydroperoxide (CHP)

The depolymerization of PMMA using CHP was conducted following the procedure established for BPO (section 2.2.1.1) with stock solutions prepared as follows:

20 mg of PMMA was dissolved in 10 mL of trichlorobenzene, and 18 μL CHP (80 wt%) was added in 5 mL of trichlorobenzene. 1 mL PMMA stock solution, and 1 mL CHP stock solution were mixed in a 20 mL Schlenk glass tube to reach the initial concentration ($[RU_0]$ = 10 mM, RU_0 : CHP = 1 : 1).

2.2.1.5 Control Experiment: Depolymerization of PMMA without Peroxides

The control experiment of depolymerization of PMMA was conducted following the procedure established for BPO (section 2.2.1.1) with stock solutions prepared as follows:

20 mg of PMMA was dissolved in 10 mL of trichlorobenzene as the reactant stock. 1 mL PMMA stock solution and 1 mL of trichlorobenzene were mixed in a 20 mL Schlenk glass tube to reach the initial concentration ($[RU_0]$ = 10 mM).

2.2.2 Peroxide Loading in Depolymerization of FRP-PMMA

2.2.2.1 Depolymerization using Different Loadings of CHP

Screening for the optimal CHP loading in the depolymerization of PMMA was conducted following the procedure established for BPO (Section 2.2.1.1), with stock solutions prepared as follows:

Reaction mixtures with varying molar equivalents were prepared by mixing stock solutions as described below. Reaction solutions with varying loadings of CHP were prepared; 0.1 eq CHP is used as a representative example. 20 mg of PMMA was dissolved in 10 mL of trichlorobenzene, and 37 μL of CHP (80 wt%) was added in 5 mL of trichlorobenzene. 1 mL PMMA stock solution, 0.05 mL CHP stock solution, and 0.95 mL trichlorobenzene were mixed in a 20 mL Schlenk glass tube to reach the initial concentrations ($[RU_0]$ = 10 mM, RU_0 : CHP = 1 : 0.1). The preparation of reaction solutions with other CHP loadings is shown in **Table S1**. The mixtures were formulated to achieve a target initial concentration of $[RU_0]$ = 10 mM at a specific molar ratio RU_0 : CHP = 1 : x.

Table S1 Reaction solution preparation for optimization of CHP loading

CHP loading (eq)	PMMA Stock Solution (mL)	CHP Stock Solution (mL)	Trichlorobenzene (mL)
0.1	1	0.05	0.95
0.3	1	0.15	0.85
0.6	1	0.3	0.7
1.0	1	0.5	0.5
2.0	1	1	0

2.2.2.2 Depolymerization using Different Loadings of DTBP

Screening for the optimal DTBP loading in the depolymerization of PMMA was conducted following the procedure established for BPO (Section 2.2.1.1), with stock solutions prepared as follows:

Reaction mixtures with varying molar equivalents were prepared by mixing stock solutions as described below. Reaction solutions with varying loadings of DTBP were prepared, 0.1 eq DTBP is used as representative example. 20 mg of PMMA was dissolved in 10 mL of trichlorobenzene, and 37 μL of DTBP was added in 5 mL of trichlorobenzene. 1 mL PMMA stock solution, 0.05 mL DTBP stock solution, and 0.95 mL trichlorobenzene were mixed in a 20 mL Schlenk glass tube to reach the initial concentrations ($[RU_0]$ = 10 mM, RU_0 : DTBP = 1 : 0.1). The preparation of reaction solutions with other DTBP loadings is shown in **Table S2**. The mixtures were formulated to achieve a target initial concentration of $[RU_0]$ = 10 mM at a specific molar ratio RU_0 : DTBP = 1 : x.

Table S2 Reaction solution preparation for optimization of DTBP loading

DTBP loading (eq)	PMMA Stock Solution (mL)	DTBP Stock Solution (mL)	Trichlorobenzene (mL)
0.1	1	0.05	0.95
0.3	1	0.15	0.85
0.6	1	0.3	0.7
1.0	1	0.5	0.5
2.0	1	1	0

2.2.3 Depolymerization of FRP-PMMA via Scheduled Dosing Methodology

2.2.3.1 Depolymerization with Scheduled Dosing DTBP

A scheduled dosing stock solution was prepared prior to use as described below. 18 μL of DTBP was added to 0.5 mL of trichlorobenzene in a 5 mL vial sealed with a rubber septum. The scheduled dosing solution was deoxygenated via nitrogen purging for 15 minutes and stored in the refrigerator before use.

The reaction mixture with an initial molar equivalent of 0.1 was first prepared by mixing the stocks as described below. 20 mg of PMMA was dissolved in 10 mL of trichlorobenzene, and 18 μL of DTBP was added in 5 mL of trichlorobenzene. 1 mL PMMA stock solution, 0.1 mL DTBP stock solution, and 0.9 mL trichlorobenzene were mixed in a 20 mL Schlenk

glass tube to reach the initial concentration ([RU_0] = 10 mM, RU_0 : DTBP = 1 : 0.1). The tube was sealed with a PTFE stopcock plug at the top and a rubber septum at the side and equipped with a magnetic stir bar. The solution in the tube reactor was deoxygenated via nitrogen purging for 15 minutes. The vessel was then submerged in a 170 °C oil bath (stirring at 400 rpm), with the internal liquid level remaining approximately 1 cm below the oil surface. The reaction was stopped at 3 and 6 h, respectively, by removing the vessel from the oil bath and cooling with water at room temperature. Subsequently, 10 μL of scheduled dosing stock solution was added to the solution, and the reactor was put back in the oil bath to resume the reaction. The reaction was quenched after 9 h by removing the vessel from the heat and cooling to room temperature.

2.2.3.2 Kinetic Experiment

Kinetic Experiments for the scheduled dosing experiment was conducted following the procedure established in section 2.2.3.1. Aliquots were periodically withdrawn under a nitrogen blanket at 0.5, 3, 6, and 9 h as shown in Scheme S2. Following each sampling event, the tubes were returned to the oil bath to continue the reaction.

Kinetic experiments (single batch) with 0.1 and 0.3 equivalents of DTBP were conducted as follows: First, a shared polymer stock solution was prepared by dissolving 20 mg of PMMA in 10 mL of trichlorobenzene. 18 μL of DTBP was added to 5 mL trichlorobenzene as the DTBP stock solution.

To prepare the 0.1 equivalent mixture ([RU_0] = 10 mM, RU_0:DTBP = 1:0.1), 1.0 mL of the PMMA stock, 0.1 mL of DTBP stock, and 0.9 mL of trichlorobenzene were mixed in a 20 mL Schlenk glass tube. For the 0.3 equivalent mixture ([RU_0] = 10 mM, RU_0:DTBP = 1:0.3), 1.0 mL of the PMMA stock, 0.3 mL of DTBP stock, and 0.7 mL of trichlorobenzene were mixed in a separate 20 mL Schlenk tube.

Each tube was sealed with a PTFE stopcock plug at the top and a rubber septum at the side, and equipped with a magnetic stir bar. The solutions were deoxygenated via nitrogen purging for 15 minutes. The vessels were then submerged in a 170 °C oil bath (stirring at 400 rpm) such that the internal liquid level remained approximately 1 cm below the oil surface. Aliquots were periodically withdrawn under a nitrogen blanket at 0.5, 3, 6, and 9

h (Scheme S2). Following each sampling event, the tubes were returned to the oil bath to continue the reaction.

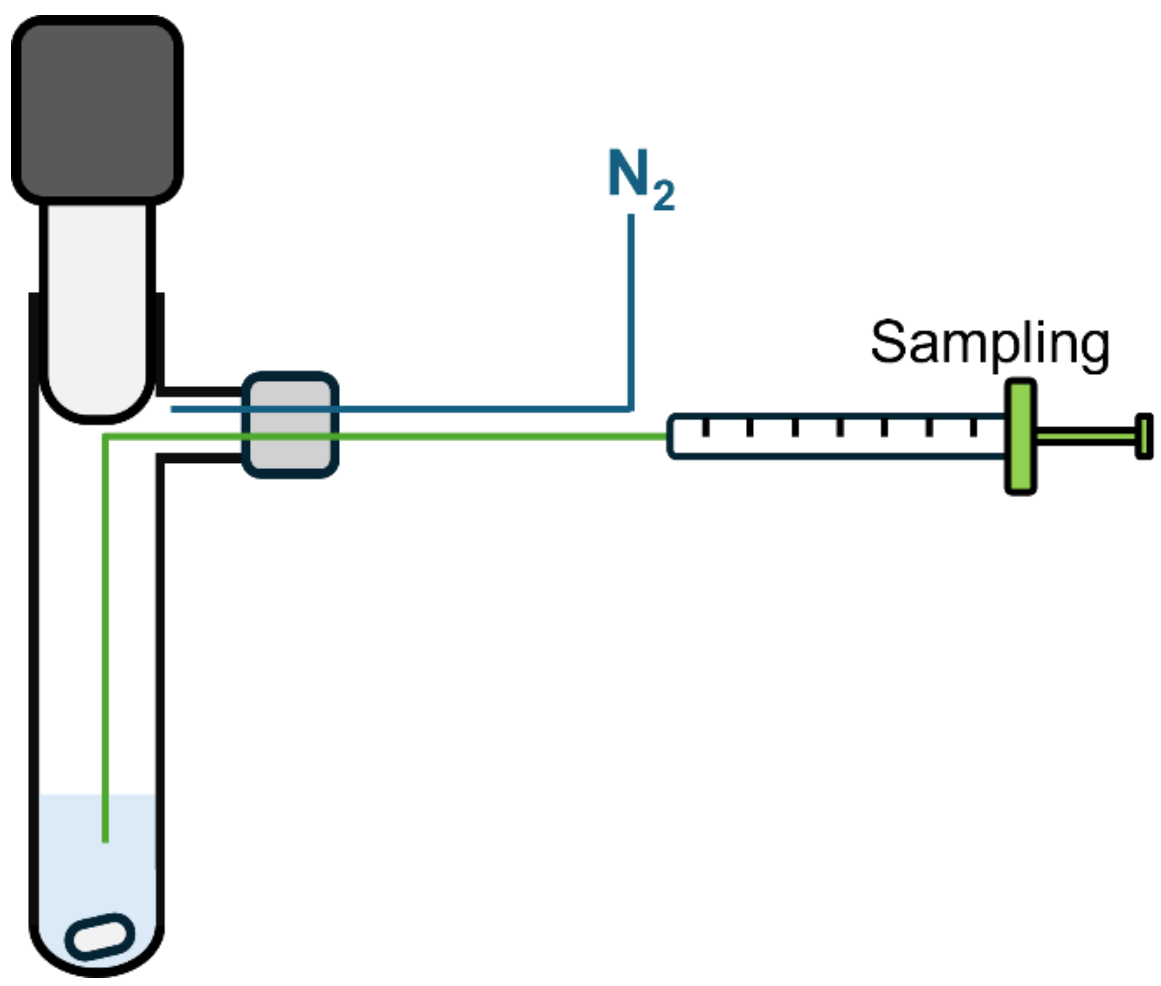

Scheme S2 Kinetic Experiment Sampling Setup

2.2.4 Depolymerization of the FRP-PMMA in Diphenyl Ether

2.2.4.1 Depolymerization with DTBP in Diphenyl Ether

Depolymerization of PMMA in diphenyl ether was conducted following the procedure established for BPO (Section 2.2.1.1), with stock solutions prepared as follows:

Reaction mixtures with varying molar equivalents were prepared by mixing stock solutions as described below. Reaction solutions with varying loadings of DTBP were prepared; 0.6 eq DTBP is used as a representative example. 20 mg of PMMA was dissolved in 10 mL of diphenyl ether, and 56 μL of DTBP was added in 5 mL of diphenyl ether. 1 mL PMMA stock solution, 0.2 mL DTBP stock solution, and 0.8 mL diphenyl ether were added in a 20 mL Schlenk glass tube to reach the initial concentrations ($[RU_0]$ = 10 mM, RU_0 : DTBP = 1 : 0.6). The preparation of reaction solutions with other DTBP loadings is shown in Table S3. The mixtures were formulated to achieve a target initial concentration of $[RU_0]$ = 10 mM at a specific molar ratio RU_0 : DTBP = 1 : x.

Table S3 Reaction solution preparation for different DTBP loadings in diphenyl ether

DTBP loading (eq)	PMMA Stock Solution (mL)	DTBP Stock Solution (mL)	Diphenyl Ether (mL)
0.6	1	0.2	0.8
1.2	1	0.4	0.6
3	1	1	0

2.2.4.2 Depolymerization with Increasing Temperature with DTBP in Diphenyl Ether

20 mg of PMMA was dissolved in 10 mL of diphenyl ether, and 11 μL of DTBP was added in 5 mL diphenyl ether. 1 mL PMMA stock solution, and 1 mL DTBP stock solution were mixed in a 20 mL Schlenk glass tube to reach the initial concentrations ([RU_0] = 10 mM, RU_0 : DTBP = 1 : 0.6). The solution in the tube reactor was deoxygenated via nitrogen purging for 15 minutes. The vessel was then submerged in a 190 or 220 °C oil bath (stirring at 400 rpm), with the internal liquid level remaining approximately 1 cm below the oil surface. The reaction was quenched after 23 h by removing the vessel from the heat and cooling to room temperature. The depolymerization analysis was performed following the procedure established for BPO (section 2.2.1.1).

2.2.4.3 Depolymerization with Increasing Temperature with CHP in Diphenyl Ether

20 mg of PMMA was dissolved in 10 mL of diphenyl ether, and 18 μL of CHP (80 wt%) was added in 5 mL of diphenyl ether. 1 mL PMMA stock solution and 1 mL CHP stock solution were mixed in a 20 mL Schlenk glass tube to reach the initial concentrations ([RU_0] = 10 mM, RU_0 : CHP = 1 : 1). The solution in the tube reactor was deoxygenated via nitrogen purging for 15 minutes. The vessel was then submerged in a 190 or 220 °C oil bath (stirring at 400 rpm), with the internal liquid level remaining approximately 1 cm below the oil surface. The reaction was quenched after 23 h by removing the vessel from the heat and cooling to room temperature. The depolymerization analysis was performed following the procedure established for BPO (section 2.2.1.1).

2.2.4.4 Depolymerization Using N-chlorosaccharin (NCSC) as Chlorine Source in Diphenyl Ether

Depolymerization of PMMA with NCSC as chlorine source in diphenyl ether was conducted following the procedure established for BPO (Section 2.2.1.1), with stock solutions prepared as follows:

20 mg of PMMA was dissolved in 10 mL of diphenyl ether, and 2.2 mg NCSC and 11 μL DTBP were added in 5 mL of diphenyl ether. 1 mL PMMA stock solution and 1 mL NCSC/DTBP stock solution were mixed in a 20 mL Schlenk glass tube to reach the initial concentrations ([RU_0] = 10 mM, RU_0 : NCSC: DTBP = 1 : 0.1 : 0.6).

2.2.4.5 Depolymerization Using Trichloroisocyanuric acid (TCCA) as Chlorine Source in Diphenyl Ether

Depolymerization of PMMA with TCCA as chlorine source in diphenyl ether was conducted following the procedure established for BPO (Section 2.2.1.1), with stock solutions prepared as follows:

20 mg of PMMA was dissolved in 10 mL of diphenyl ether, and 0.16 mg TCCA and 11 μL DTBP were added in 5 mL of diphenyl ether. 1 mL PMMA stock solution and 1 mL TCCA/DTBP stock solution were mixed in a 20 mL Schlenk glass tube to reach the initial concentrations ([RU_0] = 10 mM, RU_0 : active Cl from TCCA: DTBP = 1 : 0.1 : 0.6).

2.2.4.6 Depolymerization of PMMA with DTBP Using Co-solvent, TCB/DPE

Depolymerization of PMMA with DTBP in co-solvent TCB/DPE conducted following the procedure established for BPO (Section 2.2.1.1), with stock solutions prepared as follows:

Co-solvent TCB/DPE (50% volume/50% volume) were mixed prior to usage. 20 mg of PMMA was dissolved in 10 mL of co-solvent, and 11 μL DTBP were added in 5 mL of co-solvent. 1 mL PMMA stock solution and 1 mL DTBP stock solution were mixed in a 20 mL Schlenk glass tube to reach the initial concentrations ([RU_0] = 10 mM, RU_0 : DTBP = 1 : 0.6).

2.2.5 Depolymerization of PMMA Synthesized via FRP with Azo-Initiator

2.2.5.1 Depolymerization of the PMMA with 1,1′-Azobis(cyclohexanecarbonitrile) (ABCN) in Trichlorobenzene (TCB)

Stock solutions were prepared as described: 20 mg of PMMA was dissolved in 10 mL trichlorobenzene, and 24.4 mg ABCN was dissolved in 5 mL trichlorobenzene.

1 mL PMMA stock solution and 1 mL ABCN stock solution were mixed ([RU_0] = 10 mM, RU_0 : ABCN = 1 : 1) in a 20 mL Schlenk glass tube with a magnetic stir bar. The tube was

sealed with a PTFE stopcock plug at the top and a rubber septum at the side. The solution was deoxygenated via nitrogen purging for 15 minutes. The vessel was then submerged in a 170 °C oil bath (stirring at 400 rpm), with an internal liquid level approximately 1 cm below the oil surface. The reaction was quenched after 23 h by removing the vessel from the heat and cooling to room temperature.

A 0.35 mL aliquot was taken for 1H NMR and SEC analyses. To prepare the SEC samples, 0.3 mL solution was transferred to a 5 mL vial, dried under a stream of air, and redissolved in 0.35 mL DMAc. The resulting solution was filtered through a 0.45 μm PTFE membrane syringe filter into a vial equipped with an insert. For 1H NMR analysis, the remaining 50 μL aliquot was diluted in CDCl3.

2.2.5.2 Depolymerization Using N-chlorosaccharin (NCSC) as Chlorine Source in Diphenyl Ether

Depolymerization of PMMA with NCSC as chlorine source in diphenyl ether was conducted following the procedure established in Section 2.2.5.1, with stock solutions prepared as follows:

20 mg of PMMA was dissolved in 10 mL of diphenyl ether, and 2.2 mg NCSC and 24.4 mg ABCN were added in 5 mL of diphenyl ether. 1 mL PMMA stock solution and 1 mL NCSC/ABCN stock solution were mixed in a 20 mL Schlenk glass tube to reach the initial concentrations ([RU_0] = 10 mM, RU_0 : NCSC: ABCN = 1 : 0.1 : 1).

2.2.5.3 Depolymerization Using Trichloroisocyanuric acid (TCCA) as Chlorine Source in Diphenyl Ether

Depolymerization of PMMA with TCCA as chlorine source in diphenyl ether was conducted following the procedure established in Section 2.2.5.1, with stock solutions prepared as follows:

20 mg of PMMA was dissolved in 10 mL of diphenyl ether, and 0.16 mg TCCA and 24.4 mg ABCN were added in 5 mL of diphenyl ether. 1 mL PMMA stock solution and 1 mL TCCA/ABCN stock solution was mixed in a 20 mL Schlenk glass tube to reach the initial concentrations ([RU_0] = 10 mM, RU_0 : active Cl from TCCA: ABCN = 1 : 0.1 : 1).

2.2.6 Depolymerization of the Commercial PMMA

2.2.6.1 Depolymerization of the Commercial PMMA with Batch Condition

The blue Plexiglas was shredded prior to usage to facilitate dissolution.

The depolymerization of blue Plexiglas using DTBP as the HAT agent was conducted following the procedure established for BPO (section 2.2.1.1) with stock solutions prepared as follows:

20 mg of Plexiglas (shredded) was dissolved in 10 mL of trichlorobenzene at 60 °C overnight to make the PMMA stock solution. In a separate vial, 11 μL DTBP was added in 5 mL of trichlorobenzene. To prepare the depolymerization solution, 1 mL PMMA stock solution and 1 mL DTBP stock solution were mixed in a 20 mL Schlenk glass tube to reach the initial concentrations ($[RU_0]$ = 10 mM, RU_0 : DTBP = 1 : 0.6).

2.2.6.2 Depolymerization of the Commercial PMMA with Scheduled Dosing Condition

The blue Plexiglas was shredded prior to usage to facilitate dissolution.

20 mg of Plexiglas (shredded) was dissolved in 10 mL of trichlorobenzene at 60 °C overnight to make the PMMA stock solution. 18 μL of DTBP was added in 5 mL of trichlorobenzene as the DTBP stock solution. A DTBP scheduled dosing stock solution was prepared as shown in Table S4, and the solution was then deoxygenated via nitrogen purging for 15 minutes and stored in the refrigerator before use.

Reaction mixtures with varying scheduled dosing conditions were prepared by mixing the stocks solution. The mixtures were formulated to achieve a target initial concentration of $[RU_0]$ = 10 mM at a specific molar ratio RU_0 : DTBP = 1 : x. The reaction started with an initial DTBP of 0.1 eq was described as an example. 1 mL PMMA stock solution, 0.1 mL DTBP stock solution, and 0.9 mL trichlorobenzene were mixed in a 20 mL Schlenk glass tube to reach the initial concentration ($[RU_0]$ = 10 mM, RU_0 : DTBP = 1 : 0.1). The preparation of reaction solutions with other DTBP loadings is shown in Table S5. The tube was sealed with a PTFE stopcock plug at the top and a rubber septum at the side and equipped with a magnetic stir bar. The solution in the tube reactor was deoxygenated via nitrogen purging for 15 minutes. The vessel was then submerged in a 170 °C oil bath (stirring at 400 rpm), with the internal liquid level remaining approximately 1 cm below

the oil surface. The reaction was stopped at 3 and 6 h, respectively, by removing the vessel from the oil bath and cooling with water at room temperature. Subsequently, 10 µL of scheduled dosing stock solution was added to the solution, and the reactor was put back in the oil bath to resume the reaction. The reaction was quenched after 9 h by removing the vessel from the heat and cooling to room temperature.

Table S4 Scheduled dosing stock solution preparation for depolymerization of commercial PMMA

Sched. Dosing Stock Solution	DTBP (µL)	TCB (mL)
A	18	0.5
B	36	0.5
C	54	0.5
D	108	0.5

Table S5 Reaction solution preparation for depolymerization of commercial PMMA

Initial DTBP Loading (eq)	PMMA Stock Solution (mL)	DTBP Stock Solution (mL)	TCB (mL)	Sched. Dosing Stock Solution	Total DTBP Loading (eq)
0.1	1	0.1	0.9	A	0.3
0.2	1	0.2	0.8	B	0.6
0.3	1	0.3	0.7	C	0.9
0.6	1	0.6	0.4	D	1.8

2.2.7 Depolymerization of the Other Polymethacrylates

2.2.7.1 Depolymerization of Poly(Benzyl Methacrylate) (PBzMA)

A scheduled dosing stock solution was prepared prior to use as described below. 18 µL of DTBP was added to 0.5 mL of trichlorobenzene in a 5 mL vial sealed with a rubber septum. The scheduled dosing solution was deoxygenated via nitrogen purging for 15 minutes and stored in the refrigerator before use.

The reaction mixture with an initial molar equivalent of 0.1 was first prepared by mixing the stocks as described below. 35 mg of PBzMA was dissolved in 10 mL of trichlorobenzene, and 18 µL of DTBP was added in 5 mL of trichlorobenzene. 1 mL PBzMA stock solution, 0.1 mL DTBP stock solution, and 0.9 mL trichlorobenzene were

mixed in a 20 mL Schlenk glass tube to reach the initial concentration ($[RU_0]$ = 10 mM, RU_0 : DTBP = 1 : 0.1). The tube was sealed with a PTFE stopcock plug at the top and a rubber septum at the side and equipped with a magnetic stir bar. The solution in the tube reactor was deoxygenated via nitrogen purging for 15 minutes. The vessel was then submerged in a 170 °C oil bath (stirring at 400 rpm), with the internal liquid level remaining approximately 1 cm below the oil surface. The reaction was temporarily stopped at 3, 6, 24, 27, 30 h, respectively, by removing the vessel from the oil bath and cooling with water at room temperature. Subsequently, 10 μL of scheduled dosing stock solution was added to the solution, and the reactor was put back in the oil bath to resume the reaction. The reaction was quenched after 48 h by removing the vessel from the heat and cooling to room temperature.

2.2.7.2 Depolymerization of Poly(n-Butyl Methacrylate) (PBMA)

A scheduled dosing stock solution was prepared prior to use as described below. 18 μL of DTBP was added to 0.5 mL of trichlorobenzene in a 5 mL vial sealed with a rubber septum. The scheduled dosing solution was deoxygenated via nitrogen purging for 15 minutes and stored in the refrigerator before use.

The reaction mixture with an initial molar equivalent of 0.1 was first prepared by mixing the stocks as described below. 28 mg of PBMA was dissolved in 10 mL of trichlorobenzene, and 18 μL of DTBP was added in 5 mL of trichlorobenzene. 1 mL PBMA stock solution, 0.1 mL DTBP stock solution, and 0.9 mL trichlorobenzene were mixed in a 20 mL Schlenk glass tube to reach the initial concentration ($[RU_0]$ = 10 mM, RU_0 : DTBP = 1 : 0.1). The tube was sealed with a PTFE stopcock plug at the top and a rubber septum at the side and equipped with a magnetic stir bar. The solution in the tube reactor was deoxygenated via nitrogen purging for 15 minutes. The vessel was then submerged in a 170 °C oil bath (stirring at 400 rpm), with the internal liquid level remaining approximately 1 cm below the oil surface. The reaction was temporarily stopped at 3, 6, 24, 27, 30 h, respectively, by removing the vessel from the oil bath and cooling with water at room temperature. Subsequently, 10 μL of scheduled dosing stock solution was added to the solution, and the reactor was put back in the oil bath to resume the reaction. The reaction was quenched after 48 h by removing the vessel from the heat and cooling to room temperature.

2.3 Vapor-Trapping Experiments for Confirmation of Hydrochloric acid (HCl) formation

2.3.1 HCl Formation Confirmation under Condition of DTBP in TCB Using Acid Formation and Chloride Salt Formation Indicator

Indicator solutions were prepared as described below. 1 mg methyl red was dissolved in 1 mL ethanol as methyl red stock solution. 10 mg NaOH was dissolved in 1 L deionized water (DI water) as basic aqueous solution. 5 drops of methyl red stock solution were added in basic aqueous solution to be utilized as acid formation indicator. 0.85 g $AgNO_3$ was dissolved in 50 mL DI water to be used as chloride salt formation indicator.

DTBP (46 µL, 120 mM) was added in 2 mL trichlorobenzene in a 10 mL Schlenk glass tube with a magnetic stir bar. The tube was sealed with a rubber septum at the side and connected to a round-bottom flask (receptor flask) via a bridge. The acid formation indicator or chloride salt indicator was placed in the receptor flask where a glass tube extended from the bridge was submerged to ensure the formed HCl could contact with the indicator solution well. The solution and the whole setup were deoxygenated via nitrogen purging for 15 minutes. The glass tube was then submerged in a 170 °C oil bath (stirring at 400 rpm), with the internal liquid level remaining approximately 1 cm below the oil surface. The reaction was quenched after 1 h by removing the vessel from the heat and cooling to room temperature. During the reaction, the color change or the precipitation was monitored as the indication of pH value shifting (from basic to acidic solution) or chlorine salt formation.

2.3.2 HCl Formation Confirmation under Condition of PMMA and DTBP in TCB Using Acid Formation and Chloride Salt Formation Indicator

Indicator solutions were prepared as described in section 2.3.1.

DTBP (46 µL, 120 mM) and PMMA (40 mg, 200 mM of RUC) were added in 2 mL DPE in a 10 mL Schlenk glass tube with a magnetic stir bar. The reaction following the procedure demonstrated in section 2.2.5.1 During the reaction, the color change or the precipitation was monitored as the indication of pH value shifting (from basic to acidic solution) or chlorine salt formation.

2.3.3 HCl Formation Confirmation under Condition of DTBP and TCCA in DPE Using Acid Formation and Chloride Salt Formation Indicator

Indicator solutions were prepared as described in section 2.3.1.

DTBP (46 µL, 120 mM) and TCCA (31 mg, 200 mM active Cl) were added in 2 mL DPE in a 10 mL Schlenk glass tube with a magnetic stir bar. The reaction following the procedure demonstrated in section 2.2.5.1 During the reaction, the color change or the precipitation was monitored as the indication of pH value shifting (from basic to acidic solution) or chlorine salt formation.

2.3.4 HCl Formation Confirmation under Condition of TCB alone Using Acid Formation and Chloride Salt Formation Indicator

Indicator solutions were prepared as described in section 2.3.1.

2 mL TCB was added in a 10 mL Schlenk glass tube with a magnetic stir bar. The reaction following the procedure demonstrated in section 2.2.5.1 During the reaction, the color change or the precipitation was monitored as the indication of pH value shifting (from basic to acidic solution) or chlorine salt formation.

2.4 MMA Oligomerization

2.4.1 MMA Oligomerization with Standard Batch Condition of DTBP

21 µL of MMA was mixed with 10 mL of trichlorobenzene, and 11 µL DTBP was added in 5 mL of trichlorobenzene. 1 mL MMA stock solution and 1 mL DTBP stock solution were mixed in a 20 mL Schlenk glass tube to reach the initial concentration ($[MMA]_0$ = 10 mM, MMA : DTBP = 1 : 0.6).

The tube was sealed with a PTFE stopcock plug at the top and a rubber septum at the side. The solution was deoxygenated via nitrogen purging for 15 minutes. The vessel was then submerged in a 170 °C oil bath (stirring at 400 rpm), with the internal liquid level approximately 1 cm below the oil surface. The reaction was quenched after 23 h by removing the vessel from the heat and cooling to room temperature.

A 0.35 mL aliquot was taken for ^{1}H NMR and SEC analyses. To prepare the SEC samples, 0.3 mL solution was transferred to a 5 mL vial, dried under a stream of air, and redissolved in 0.35 mL DMAc. The resulting solution was filtered through a 0.45 µm PTFE membrane

syringe filter into a vial equipped with an insert. For ^{1}H NMR analysis, the remaining 50 μL aliquot was diluted in $CDCl_3$.

3 Additional Data

3.1 Characterization of PMMA Synthesized via FRP

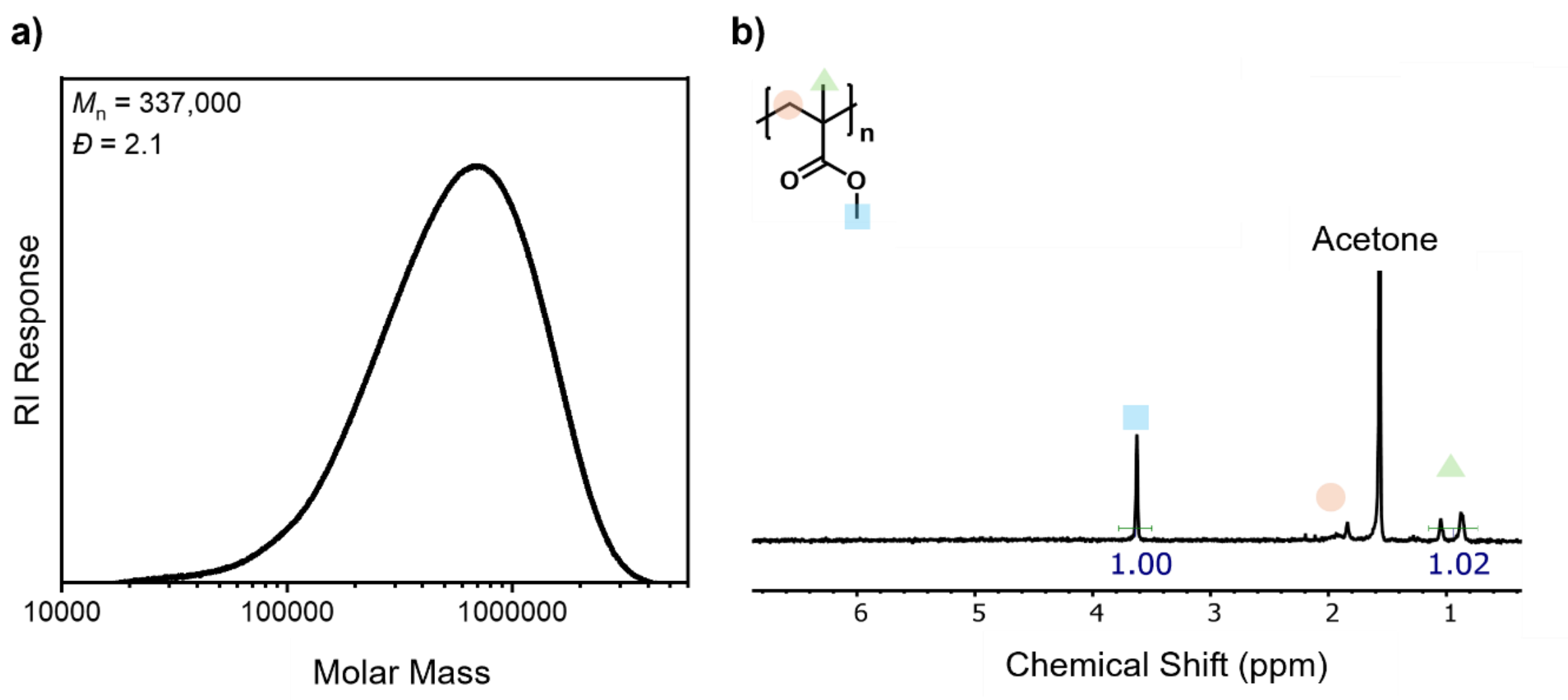

Figure S1 Characterization of PMMA synthesized via FRP: a) SEC trace (M_n = 337,000, $Đ$ = 2.1), b) ^{1}H NMR spectrum after purification.

3.2 Control Experiments – Depolymerization without Peroxide

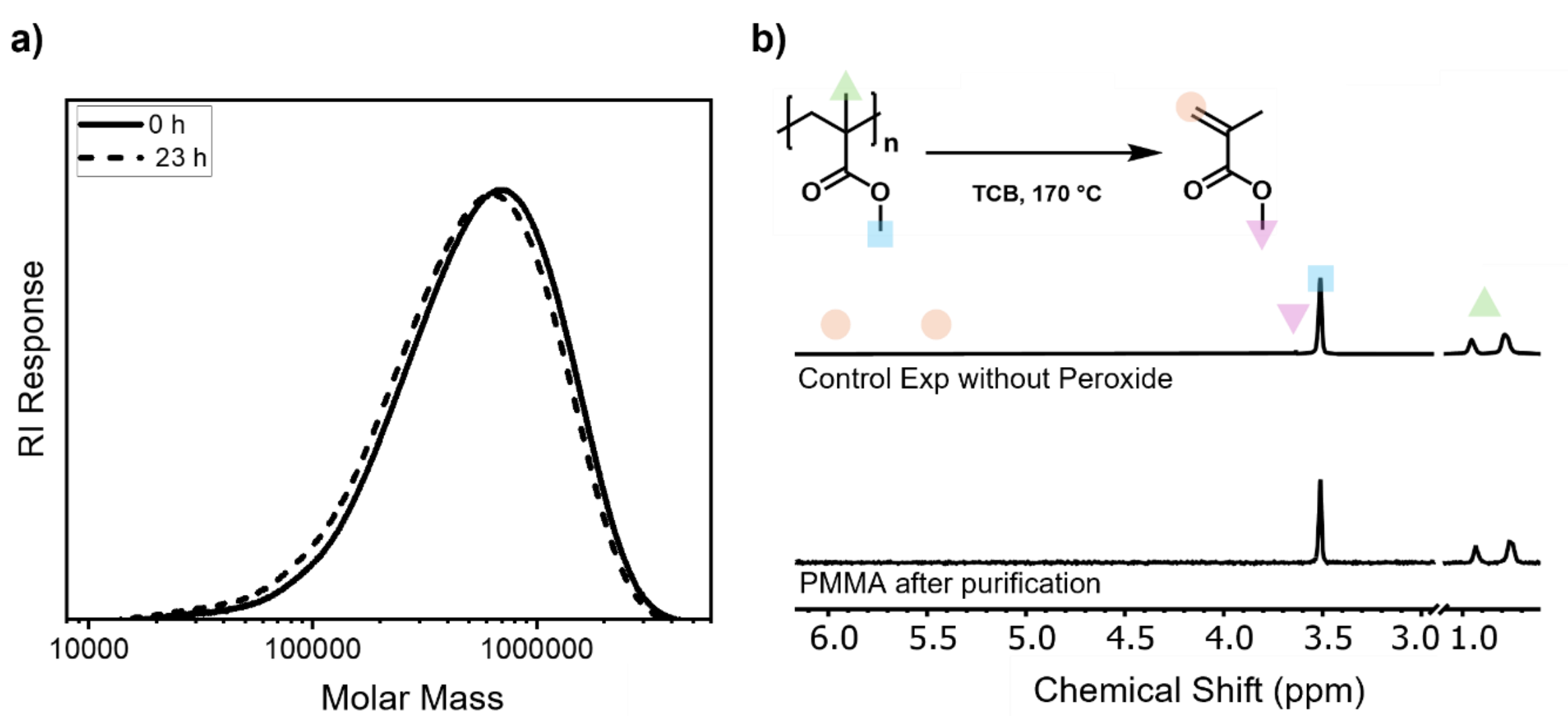

Figure S2 Control experiment with trichlorobenzene (TCB). RUC of 10 mM PMMA in TCB was reacted for 23 h at 170 °C a) SEC traces of t_{0h} and t_{23h}. b) ^{1}H NMR showed a negligible amount of monomer recovery.

3.3 Peroxide Screening for Depolymerization of PMMA

Table S6 1 h half-life temperatures for peroxides.

Peroxides	BPO	DCP	DTBP	CHP
$T_{1/2,\ 1h}$	92 °C [1]	136 °C[1]	156 °C [2]	166 °C [1]

Table S7 Depolymerization conversions determined by NMR and SEC analysis§

Peroxides	Depolymerization (%)*	SEC Conversion (%)†	M_n Shift (%)‡
No Peroxide (TCB)	1	0	0
BPO	15	18	92
DCP	56	57	82
DTBP	66	96	98
CHP	79	86	97

It is noted that calculating conversion by SEC may not be reliable due to the formation of oligomers for this work, and as such all our reactions were analyzed primarily by NMR spectroscopy.

§ RUC of 10 mM PMMA and 1 eq peroxide (RU_0: Peroxide = 1:1) in trichlorobenzene were reacted for 23 h at 170 °C.

*Depolymerization conversion was calculated as shown in **Figure S4a**

†SEC depolymerization conversions were calculated via SEC analysis, using the following formula: Conversion = $(1-(Area_t/Area_{t0}))*100$, where $Area_{t0}$ and $Area_t$ denote samples taken at time 0 h and x h.

‡ M_n Shifts were calculated via SEC analysis, using the following formula: Conversion = $((M_{nt0}-M_{nt})/ M_{nt0})*100$, where M_{nt0} and M_{nt} denote samples taken at time 0 h and x h.

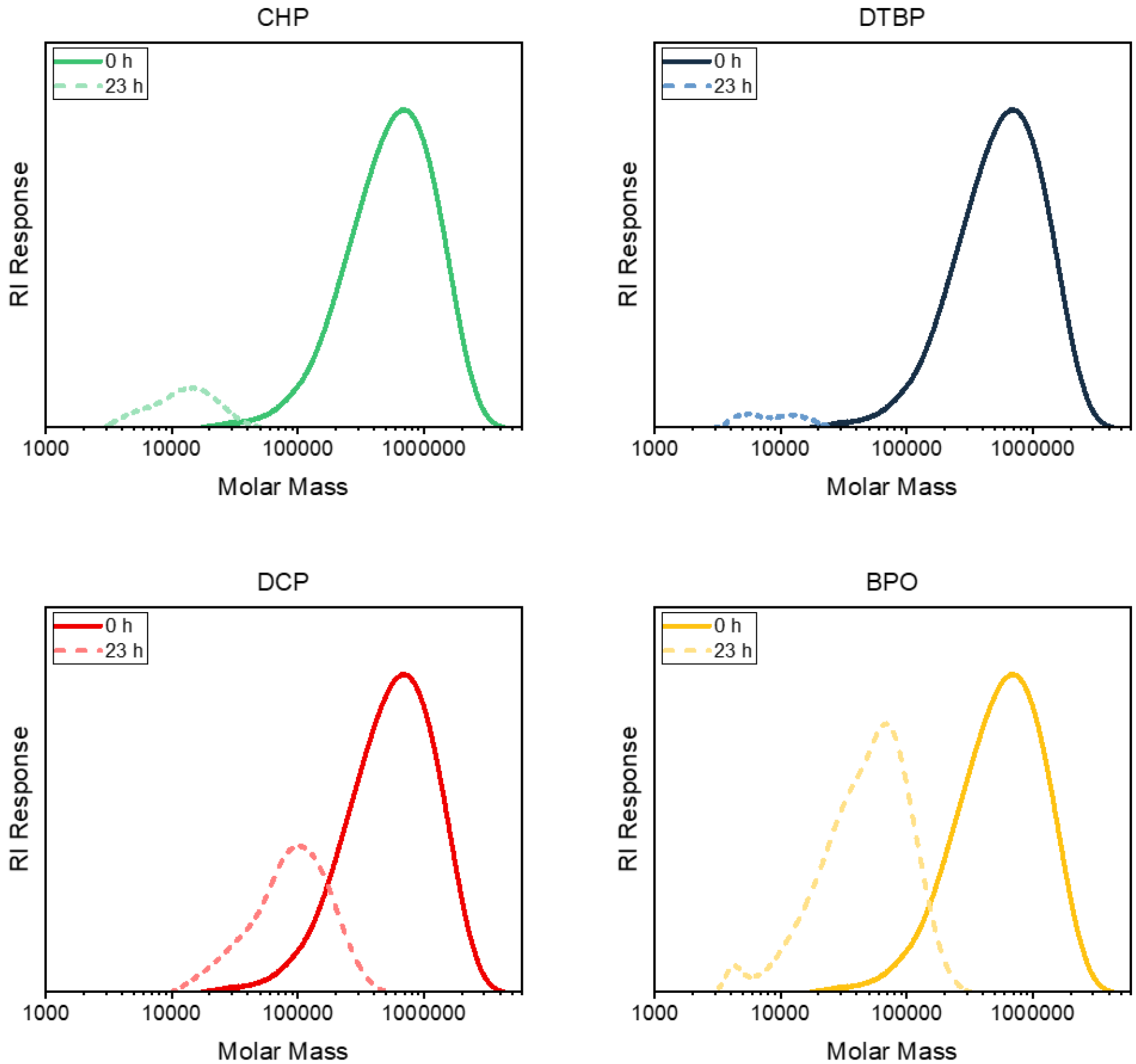

Figure S3 SEC traces of PMMA depolymerization using various peroxides. Reaction conditions: PMMA (10 mM RUC) and peroxide (1.0 eq.) in TCB at 170 °C for 23 h, showing concurrent chain scission and depolymerization.

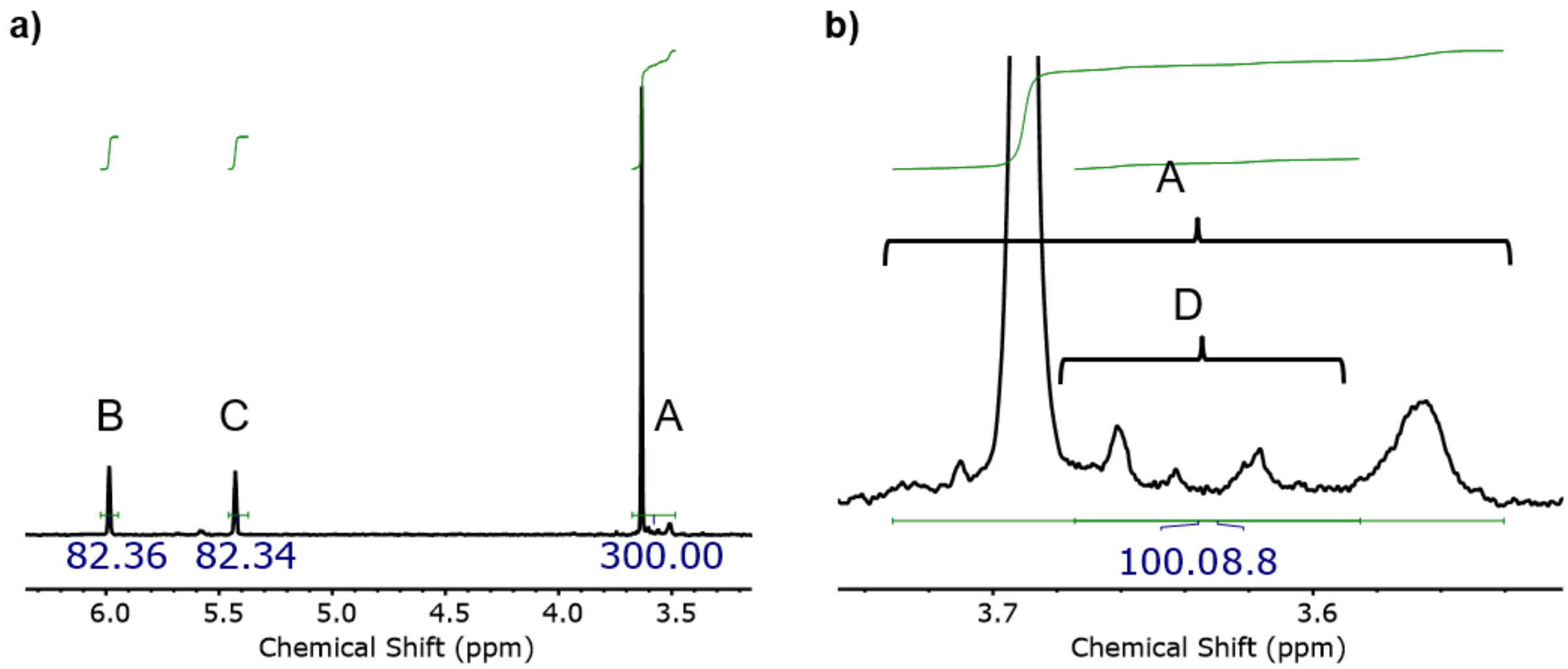

Integral area A was set to 300 first.

$$\text{Depolymerization Conversion} = \left(\frac{B + C}{2}\right)\%$$

Integral area A was set to 100 first.

$$\text{Oligomer Amount} = D\%$$

Figure S4 Example of determination of a) depolymerization conversion by NMR analysis, where in this case, depolymerization conversion is 82.35%; and b) oligomer amount by NMR analysis, where in this case, oligomer amount is 8.8%.

3.4 Optimization of Peroxides Loadings

Table S8 Evolution of depolymerization conversion and oligomer percentage determined by NMR analysis for the depolymerization of PMMA using X eq (where X = 0.1-2 eq) CHP§

Peroxide loading (eq)	Depolymerization (%)*	Oligomer Amount (%)†
1:0.1	33	1.2
1:0.3	47	1.4
1:0.6	54	3.8
1:1	79	8.9
1:2	40	10.8

§ RUC of 10 mM PMMA and X eq CHP (RU_0: CHP = 1: X, X = 0.1-2) in trichlorobenzene were reacted for 23 h at 170 °C.

*Depolymerization conversion was calculated as shown in **Figure S4a**.

†Oligomer amount was calculated as shown in **Figure S4b**.

Table S9 Evolution of depolymerization conversion and oligomer percentage determined by NMR analysis for the depolymerization of PMMA using X eq (where X = 0.1-2 eq) DTBP§

Peroxide loading (eq)	Depolymerization (%)*	Oligomer Amount (%)†
1:0.1	78	2.1
1:0.3	83	10.3
1:0.6	86	12.9
1:1	66	16.3
1:2	55	28.3

§ RUC of 10 mM PMMA and X eq DTBP (RU_0: DTBP = 1: X, X = 0.1-2) in trichlorobenzene were reacted for 23 h at 170 °C.

*Depolymerization conversion was calculated as shown in **Figure S4a**.

†Oligomer amount was calculated as shown in **Figure S4b**.

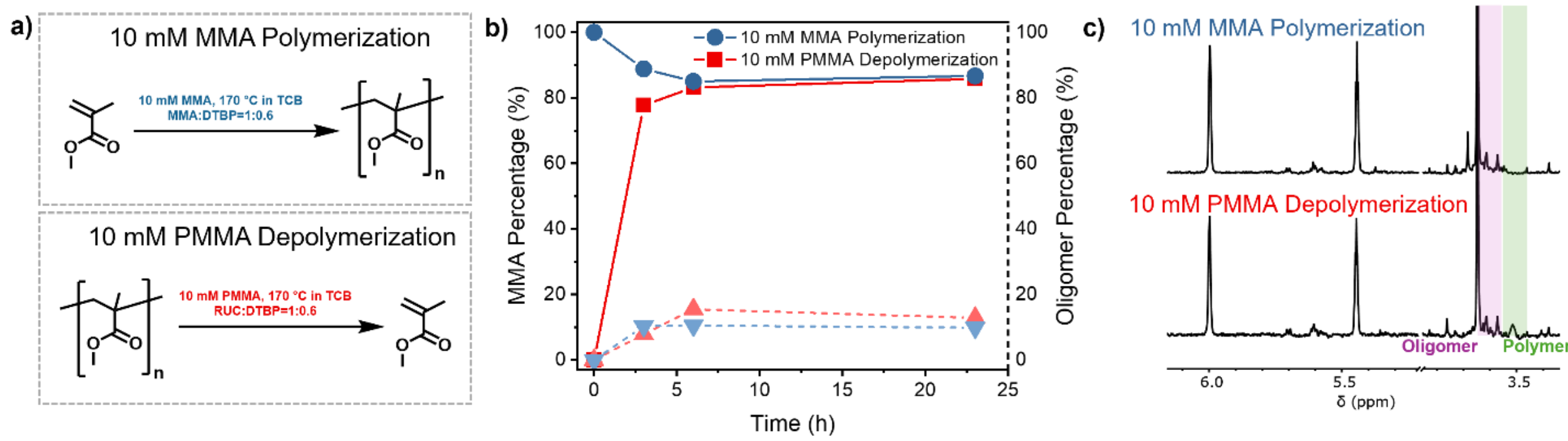

Figure S5 Thermodynamic feasibility of oligomer formation with 10 mM MMA. a) Reaction schemes MMA polymerization vs. PMMA depolymerization under identical conditions ($[RUC]_0$ or $[MMA]_0$: DTBP = 1 : 0.6, 170 °C, 23 h); b) evolution of MMA and oligomer content; c) ^1H NMR product analysis demonstrating oligomer formation with 10 mM MMA, without high-molecular-weight polymer formation.

3.5 Kinetic Profile of the Depolymerization with or without Scheduled Dosing Strategy

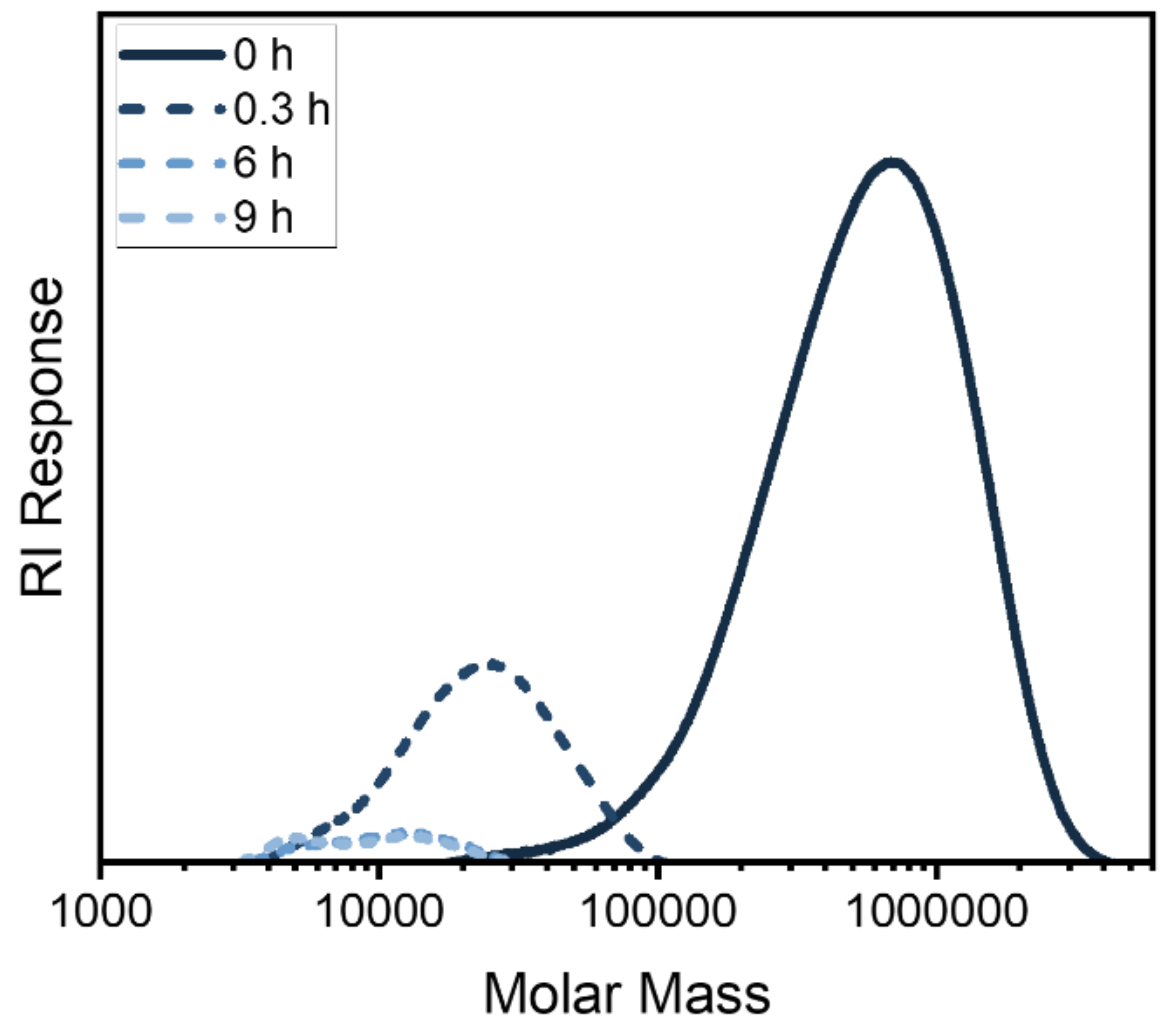

Figure S6 Evolution of molecular weight distributions during the DTBP-mediated depolymerization of PMMA, as monitored by SEC. RUC of 10 mM PMMA and 0.6 eq DTBP in trichlorobenzene was reacted for 9 h at 170 °C, as significant concurrent chain scission and depolymerization happened in the reaction.

Table S10 Depolymerization conversion determined by NMR and SEC, and M_n and M_n shift compared to the initial polymer.§

Time (h)	Depolymerization (%)*	M_n	SEC Conversion (%)†	M_n Shift (%)‡
0	0	337,000	0	0
0.3	61	17,700	74	95
6	78	8,900	96	97
9	83	7,800	96	98

It is noted that calculating conversion by SEC may not be reliable due to the formation of oligomers for this work, and as such all our reactions were analyzed primarily by NMR spectroscopy.

§ RUC of 10 mM PMMA and 0.6 eq DTBP (RU_0: DTBP = 1:0.6) in trichlorobenzene were reacted for 9 h at 170 °C, and in-situ sampling was conducted at 0.3, 6, 9 h during the reaction.

Depolymerization conversion was calculated as shown in **Figure S4a

†SEC depolymerization conversions were calculated via SEC analysis, using the following formula: Conversion = $(1-(Area_t/Area_{t0}))*100$, where $Area_{t0}$ and $Area_t$ denote samples taken at time 0 h and x h.

‡ M_n Shifts were calculated via SEC analysis, using the following formula: Conversion = $((M_{nt0}-M_{nt})/ M_{nt0})*100$, where M_{nt0} and M_{nt} denote samples taken at time 0 h and x h.

Table S11 Depolymerization conversions and oligomer percentage determined by NMR analysis for the depolymerization of PMMA using 0.1 eq DTBP.§

Time (h)	Depolymerization (%)*	Oligomer Amount (%)†
0	0	0
0.3	33	0.6
3	76	0.6
6	78	0.6
9	78	0.5

§ RUC of 10 mM PMMA and 0.1 eq DTBP (RU_0: DTBP = 1: 0.1) in trichlorobenzene were reacted for 9 h at 170 °C.

*Depolymerization conversion was calculated as shown in **Figure S4a**.

†Oligomer amount was calculated as shown in **Figure S4b**.

Table S12 Depolymerization conversions and oligomer percentage determined by NMR analysis for the depolymerization of PMMA using 0.3 eq DTBP.§

Time (h)	Depolymerization (%)*	Oligomer Amount (%)†
0	0	0
0.3	50	5.3
3	77	9.0
6	80	9.3
9	83	9.4

§ RUC of 10 mM PMMA and 0.3 eq DTBP (RU_0: DTBP = 1: 0.3) in trichlorobenzene were reacted for 9 h at 170 °C.

*Depolymerization conversion was calculated as shown in **Figure S4a**.

†Oligomer amount was calculated as shown in **Figure S4b**.

Table S13 Depolymerization conversions and oligomer percentage determined by NMR analysis for the depolymerization of PMMA using 0.3 eq DTBP with scheduled dosing strategy (initially 0.1 eq and subsequent 0.1 eq additions at 3 and 6 hrs).§

Time (h)	Depolymerization (%)*	Oligomer Amount (%)†
0	0	0
0.3	37	0.6
3	74	0.4
6	84	0.5

9	96	0.5

§ RUC of 10 mM PMMA with dosing 0.3 eq (in total) DTBP (RU_0: DTBP = 1: 0.3 (in total)) in trichlorobenzene were reacted for 9 h at 170 °C.

*Depolymerization conversion was calculated as shown in **Figure S4a**.

†Oligomer amount was calculated as shown in **Figure S4b**.

3.6 Additional Choice of Solvents for HAT Reaction-Triggered Depolymerization

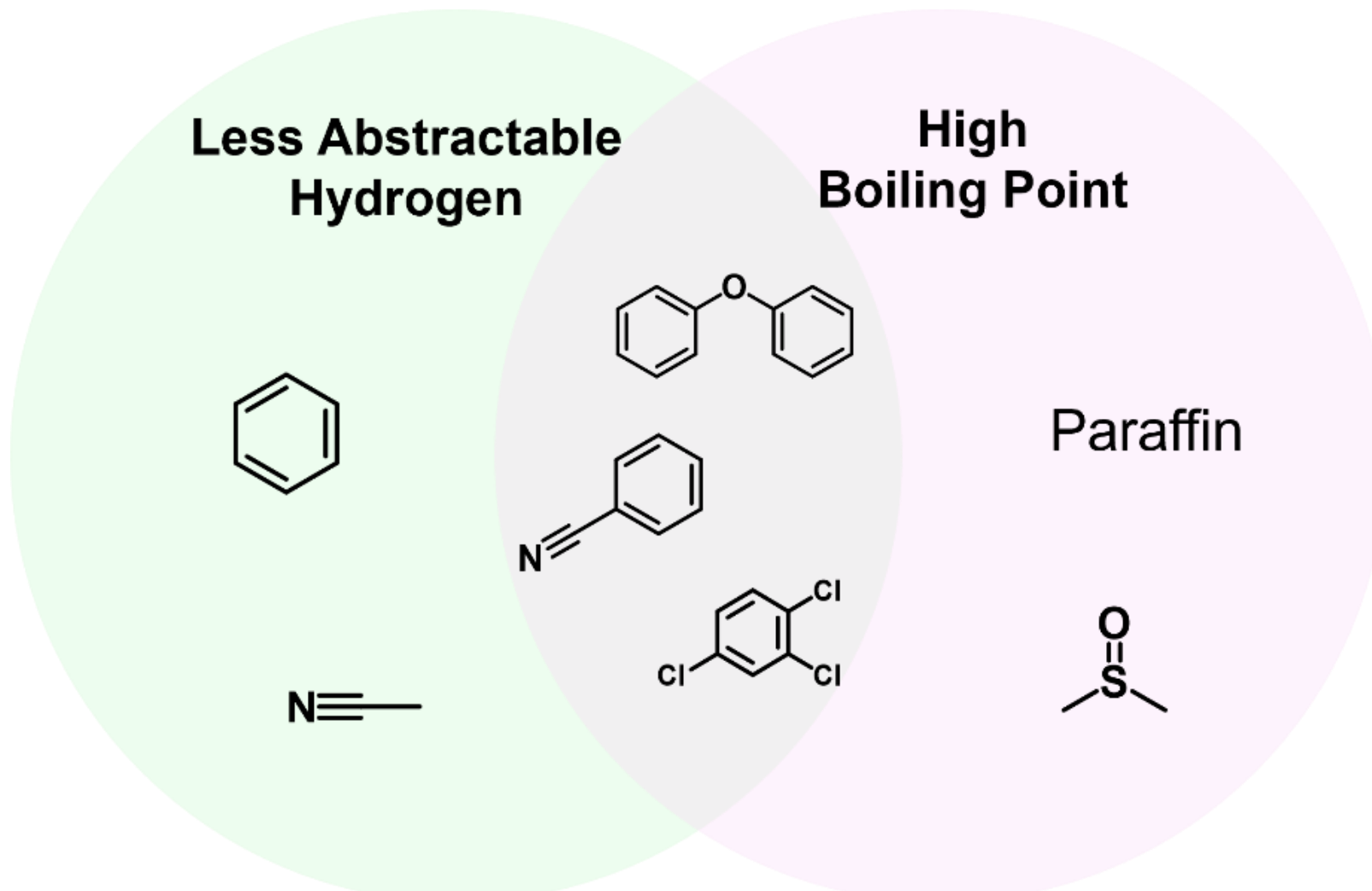

Figure S7 Venn diagram for solvent selection

3.7 Depolymerization of PMMA in non-Chlorinated Solvent, Diphenyl Ether (DPE)

Table S14 Depolymerization conversions determined by NMR analysis for the depolymerization of PMMA using varying equivalents of DTBP in DPE at 170 °C.§

Peroxide Loading (eq)	Depolymerization (%)*
1:0.6	15
1:1.2	22
1:3	25

§ RUC of 10 mM PMMA with X eq DTBP (RU_0: DTBP = 1: X, as X = 0.6-3) in DPE were reacted for 23 h at 170 °C.

*Depolymerization conversion was calculated as shown in **Figure S4a**.

Table S15 Depolymerization conversions determined by NMR analysis for the depolymerization of PMMA using DTBP in DPE at different temperatures.§

Peroxide Loading (eq)	Temperature (°C)	Depolymerization (%)*
1:0.6	190	18
1:0.6	220	45

§ RUC of 10 mM PMMA and 0.6 eq DTBP (RU_0: DTBP = 1:0.6) in DPE were reacted for 23 h at 190 and 220 °C, respectively.

*Depolymerization conversion was calculated as shown in **Figure S4a**.

Table S16 Depolymerization conversions determined by NMR analysis for the depolymerization of PMMA using CHP in DPE at different temperatures.§

Peroxide Loading (eq)	Temperature (°C)	Depolymerization (%)*
1:1	170	10
1:1	190	14
1:1	220	51

§ RUC of 10 mM PMMA and 1 eq CHP (RU_0: CHP = 1:1) in DPE were reacted for 23 h at 170, 190, 220 °C, respectively.

*Depolymerization conversion was calculated as shown in **Figure S4a**.

Table S17 Depolymerization conversions determined by NMR analysis for the depolymerization of PMMA using DTBP in DPE with external chlorine sources.

Chlorine Source	Peroxide	Depolymerization (%)*
No Chlorine Source	DTBP	15
N-chlorosaccharin (NCSC)§	DTBP	42
Trichloroisocyanuric acid (TCCA)§	DTBP	45
Trichlorobenzene†	DTBP	57

§ RUC of 10 mM PMMA, 0.6 eq DTBP, and 0.1 eq chlorine donor (RU_0: DTBP: active Cl on Chlorine Donor = 1:0.6:0.1) in DPE were reacted for 23 h at 170 °C.

† RUC of 10 mM PMMA and 0.6 eq DTBP in co-solvent (DPE/TCB(V/V)=1/1) were reacted for 23 h at 170 °C.

*Depolymerization conversion was calculated as shown in **Figure S4a**.

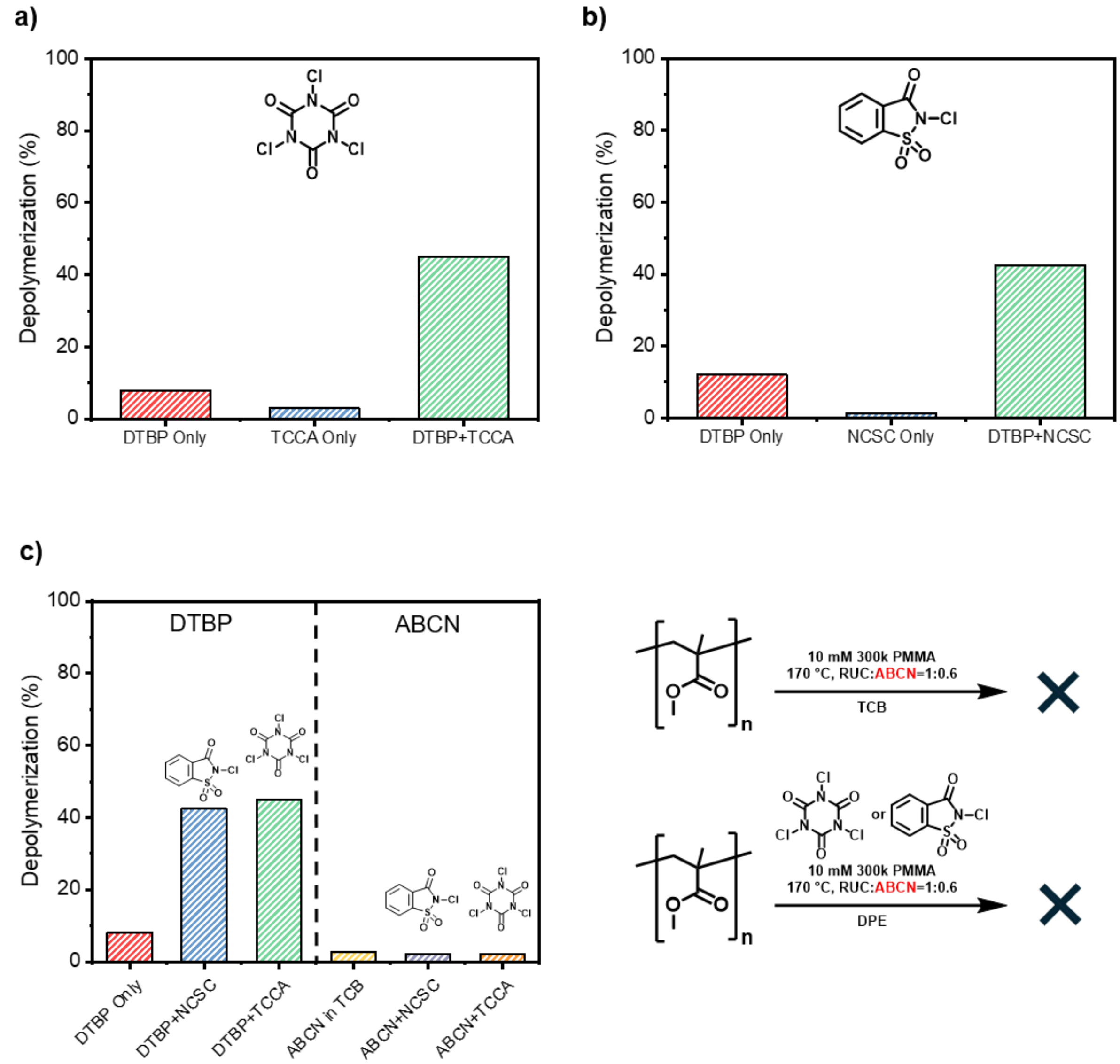

Figure S8 Control experiments evaluating PMMA depolymerization with external chlorine sources. **a)** Depolymerization using DTBP alone, TCCA alone, and a combination of DTBP and TCCA. **b)** Depolymerization using DTBP alone, NCSC alone, and a combination of DTBP and NCSC. **c)** Comparison of different thermal initiators (DTBP vs. ABCN) for PMMA depolymerization (Reaction conditions: RUC of 10 mM PMMA, 0.6 eq DTBP or 1 eq ABCN, and 0.1 eq chlorine donor at 170 °C in DPE).

3.8 Proposed Mechanism of Mid-Chain Depolymerization of PMMA via Dual-Activation Pathway

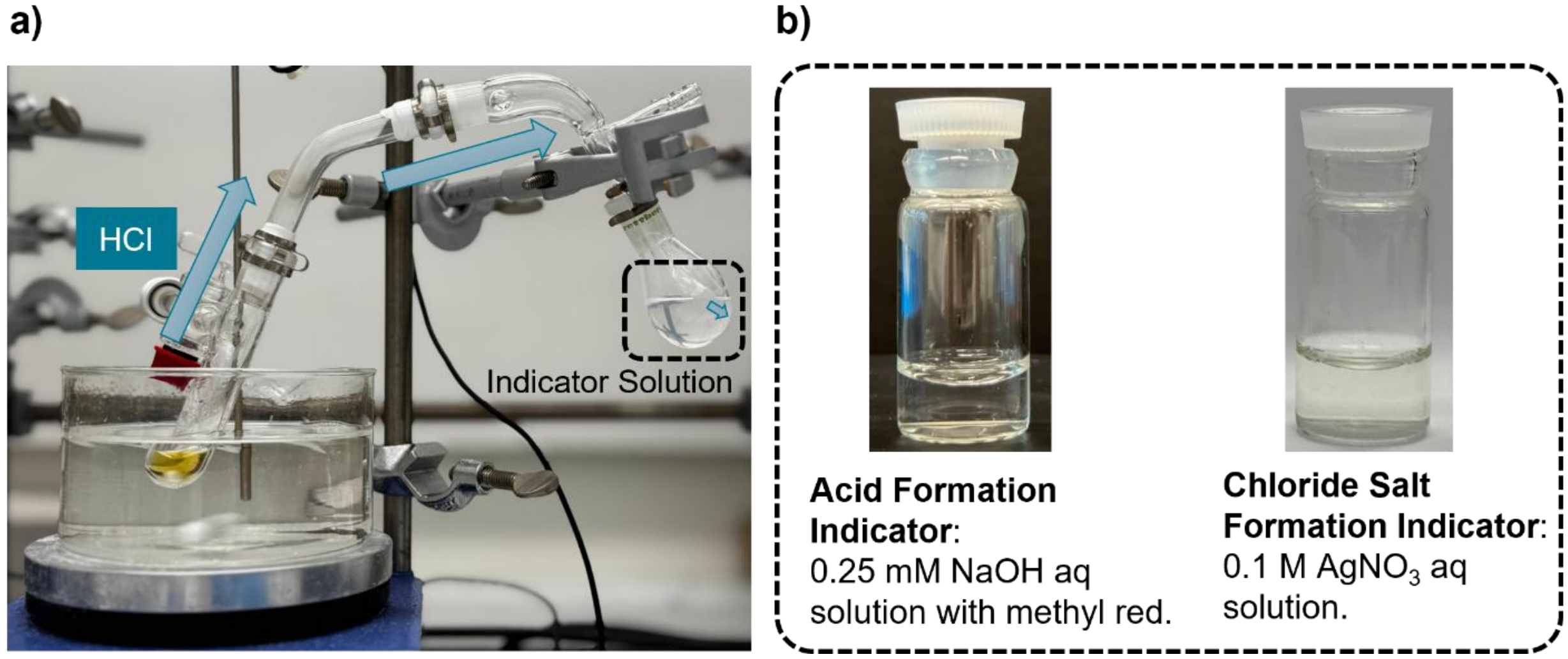

Figure S9 a) Experimental setup for vapor trapping used to confirm HCl generation. b) Compositions of indicator solutions for acid detection (yellow at basic pH and red at acidic pH) and chloride detection (AgCl precipitation in the presence of Cl^- ions).

Table S18 Hydrochloric acid formation confirmed by vapor-trapping experiments.

Solvent	Peroxide (and Chlorine Source)	Hydrogen Donor	Acid Formation Indicator[a]	Chloride Salt Formation Indicator[b]	HCl Formation
TCB[§]	DTBP	--	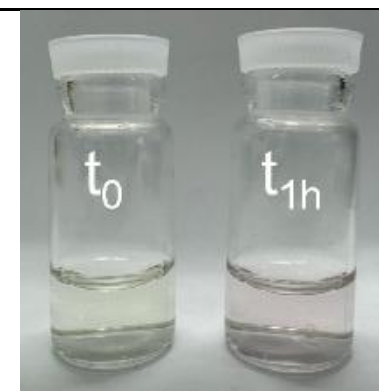	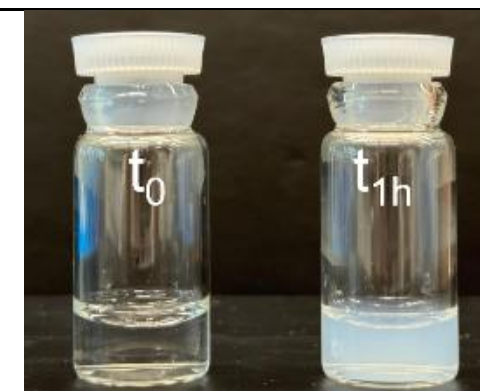	Formed
TCB	DTBP	PMMA[◊]	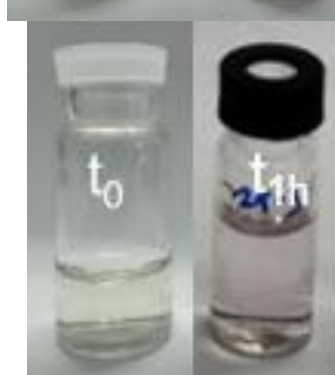	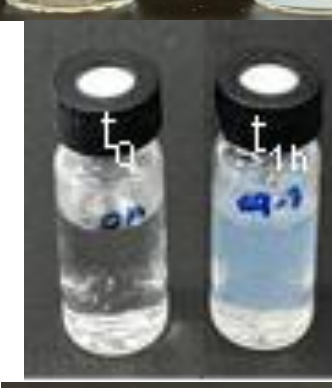	Formed
DPE[†]	DTBP and TCCA	--	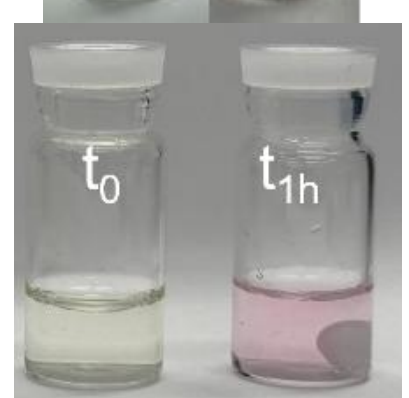	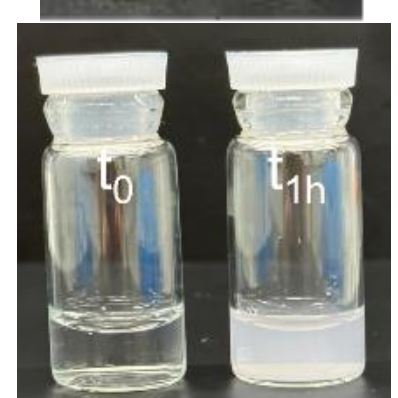	Formed
TCB	-- [‡]	--	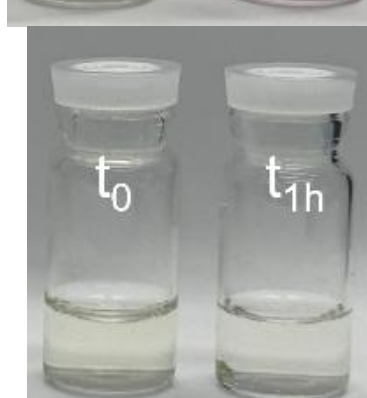	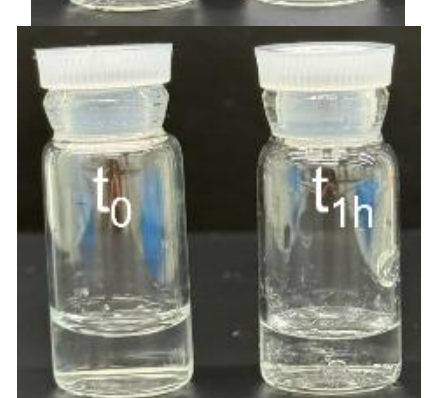	None
TCB	ABCN[♦]	--	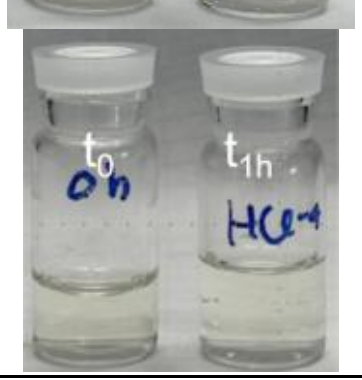	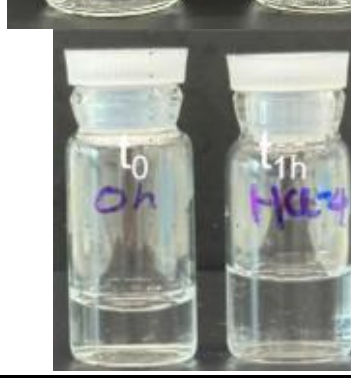	None

§ 120 mM DTBP was reacted in trichlorobenzene for 1 h at 170 °C.

◊ 120 mM DTBP and 200 mM PMMA were reacted in trichlorobenzene for 1 h at 170 °C.

† 120 mM DTBP and 200 mM TCCA were reacted in trichlorobenzene for 1 h at 170 °C.

‡ Trichlorobenzene was heated for 1 h at 170 °C as control experiment.

♦ 100 mM ABCN was reacted in trichlorobenzene for 1 h at 170 °C.

[a] Confirmed through yellow to red color transformation upon changing from basic pH to acidic pH

[b] Confirmed through AgCl precipitation

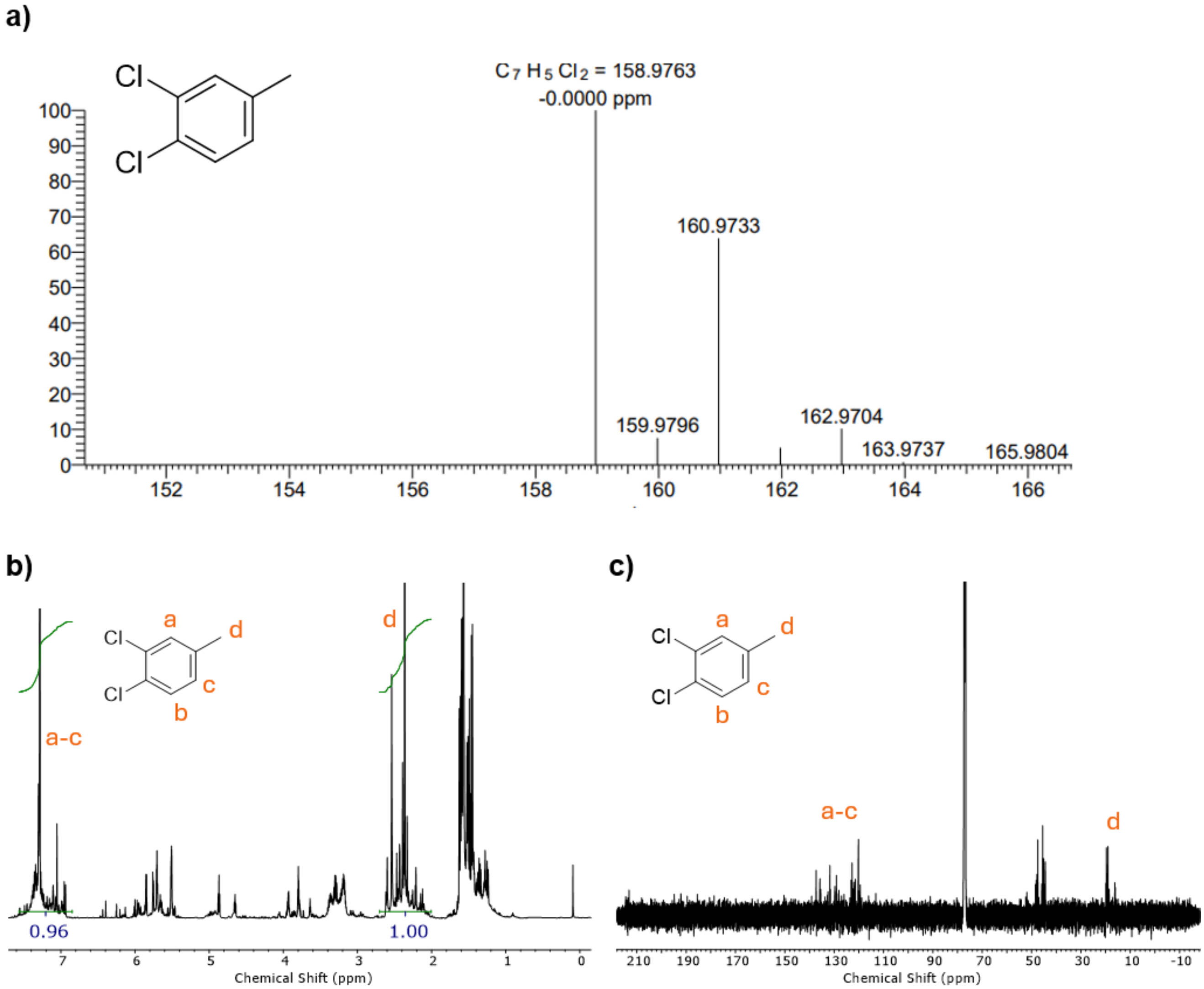

Figure S10 a) GC-MS of the major nonvolatile byproduct formed after heating DTBP in TCB at 170 °C for 15 h. b) ^{1}H NMR of the major nonvolatile byproduct formed after heating DTBP in TCB at 170 °C for 15 h. c) ^{13}C NMR of the major nonvolatile byproduct formed after heating DTBP in TCB at 170 °C for 15 h.

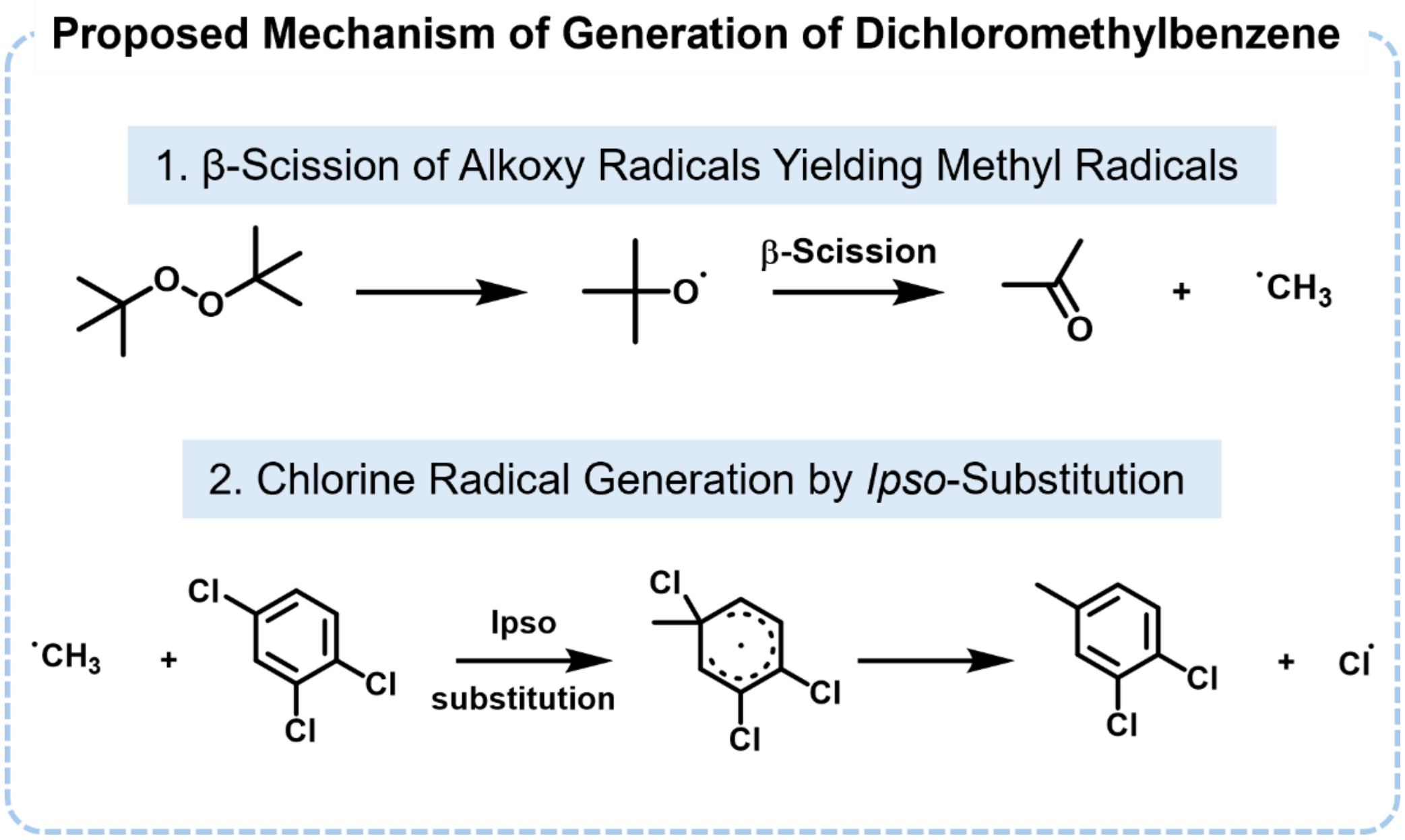

Figure S11 Possible pathway of the formation of dichloromethylbenzene during the reaction of DTBP in TCB.

3.9 Radical Electrophilicity and Bond Dissociation Energies

Table S19 Electrophilicity of chlorine radical and alkoxyl radical or hydroxyl radical derived from peroxide species.[3]

Radical	Electrophilicity (eV)
Cl·	3.8
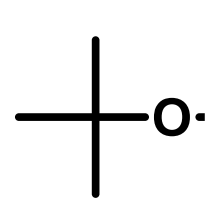	2.08†
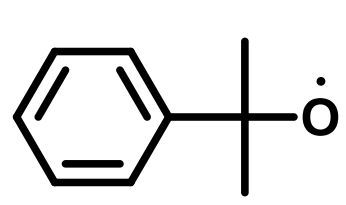	2.1-2.25†

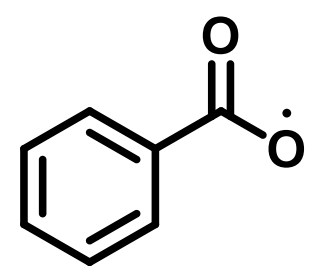	2.95†
$\dot{O}H$	2.81†

†Electrophilicity of alkoxyl radical or hydroxyl radical derived from peroxide species.[3]

Table S20 Comparison of C–H bond dissociation energies (BDE) across diverse chemical environments.[4]

C-H Bond	Bond Dissociation Energy (kcal/mol)
(C_6H_5)-H [b]	113
**H**-CH_2CH_3 [a]	101
**H**-$CH(CH_3)_2$ [a]	98
H-Cl [c]	103
H-OR [abc]	104

[a] BDE of H-OR is larger than BDE of H-CH_2CH_3 and H-$CH(CH_3)_2$, showing the potential of oxygen-centered radicals abstracting hydrogens from methyl and methylene units.
[b] BDE of (C_6H_5)-H is much larger than BDE of H-CH_2CH_3, H-$CH(CH_3)_2$, and H-OR, implying that the hydrogens on aryl rings may be less abstractable than the ones on methyl and methylene units to oxygen-centered radicals.
[c] BDE of H-OR is slightly higher than BDE of H-Cl, indicating that oxygen-centered radicals might possess the capability of conducting HAT reaction similar to chlorine-centered radicals.

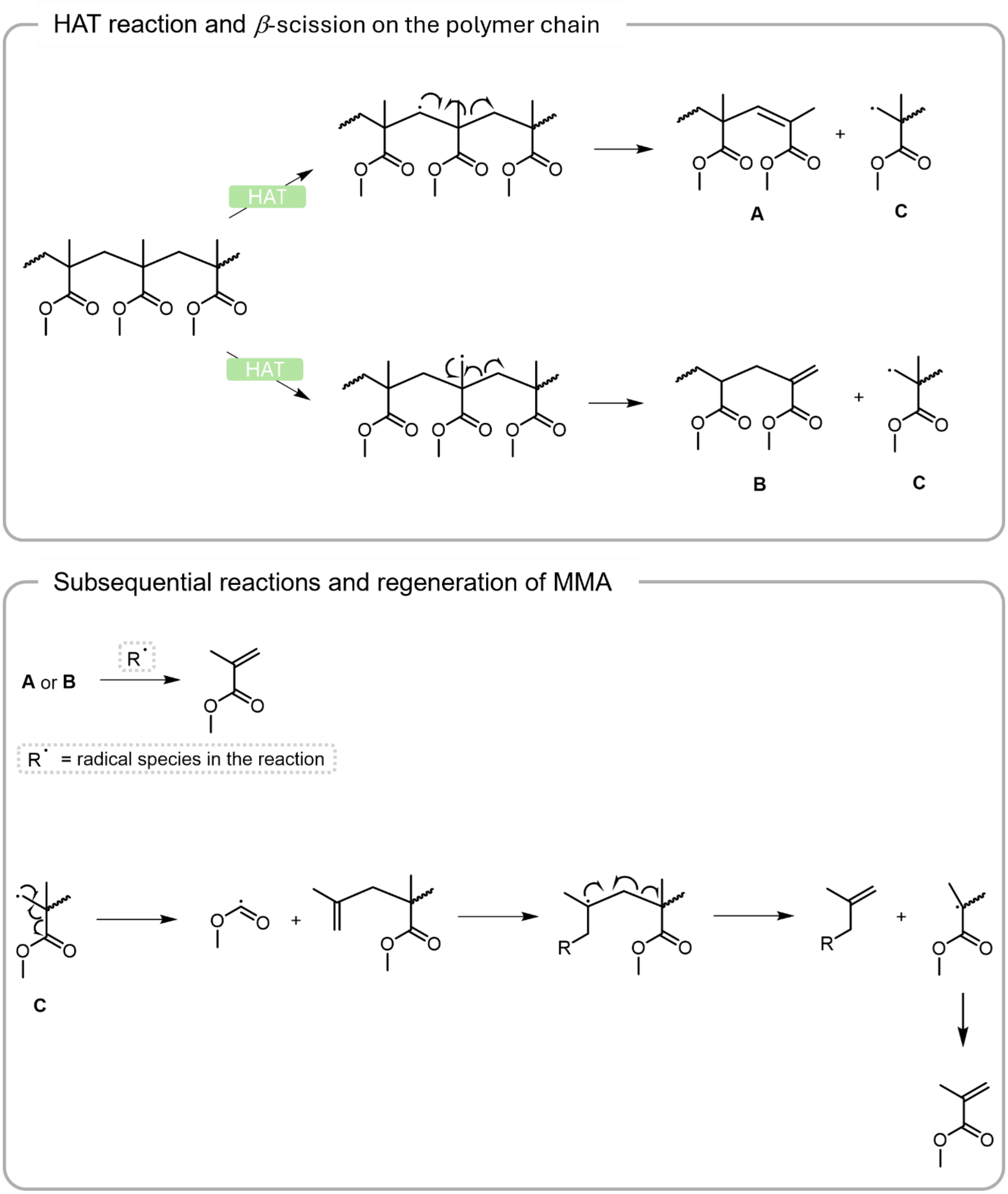

Figure S12 Proposed mechanism for mid-chain scission and depolymerization via alkoxy radical and chlorine radical.

3.10 Depolymerization of Commercial PMMAs

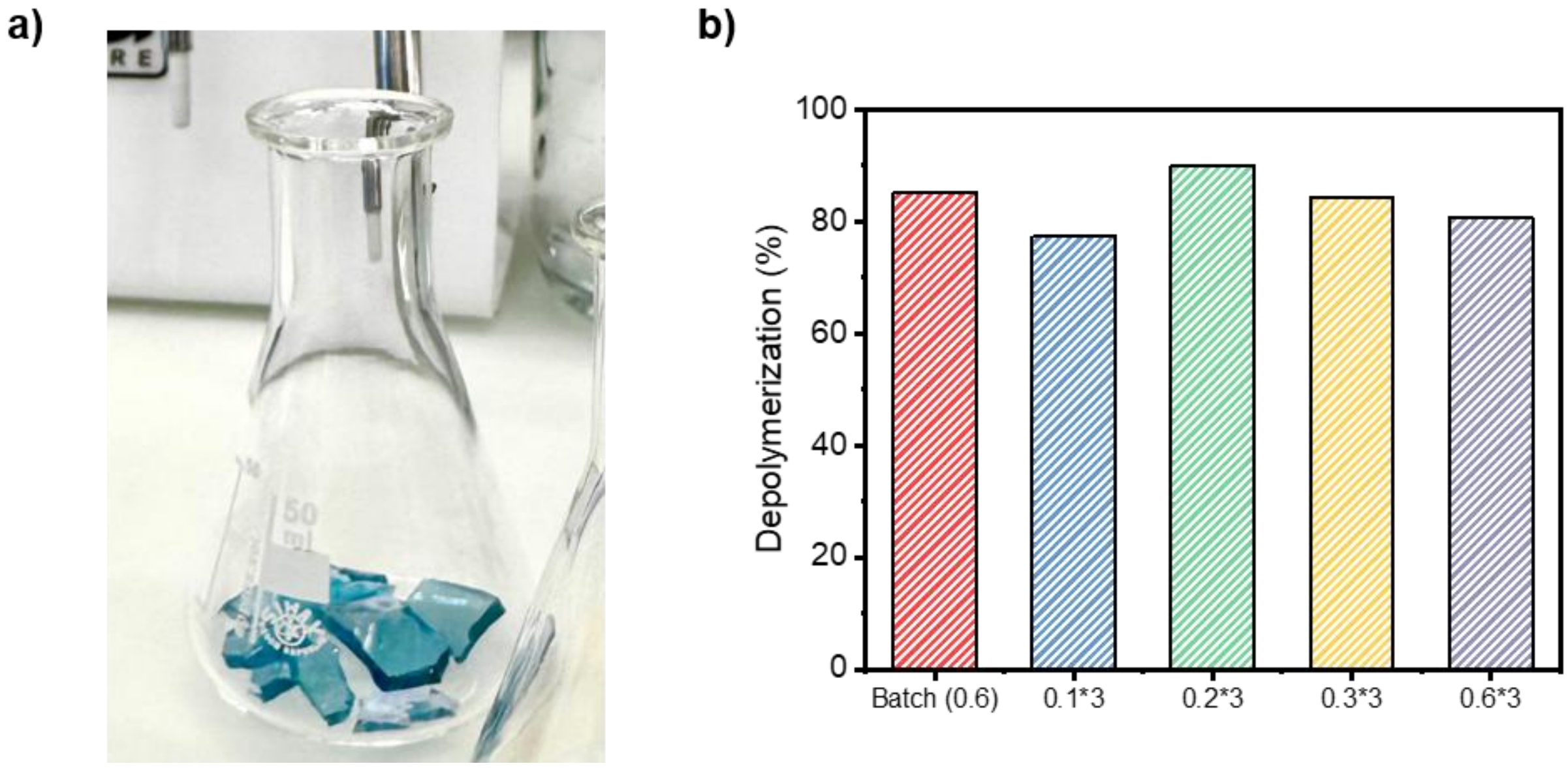

Figure S13 a) Photograph of blue Plexiglas pieces used in the demonstration of commercial PMMA depolymerization. b) Depolymerization of blue Plexiglas under various reaction conditions, as determined by ^{1}H NMR spectroscopy.

3.11 Depolymerization of Other Polymethacrylates

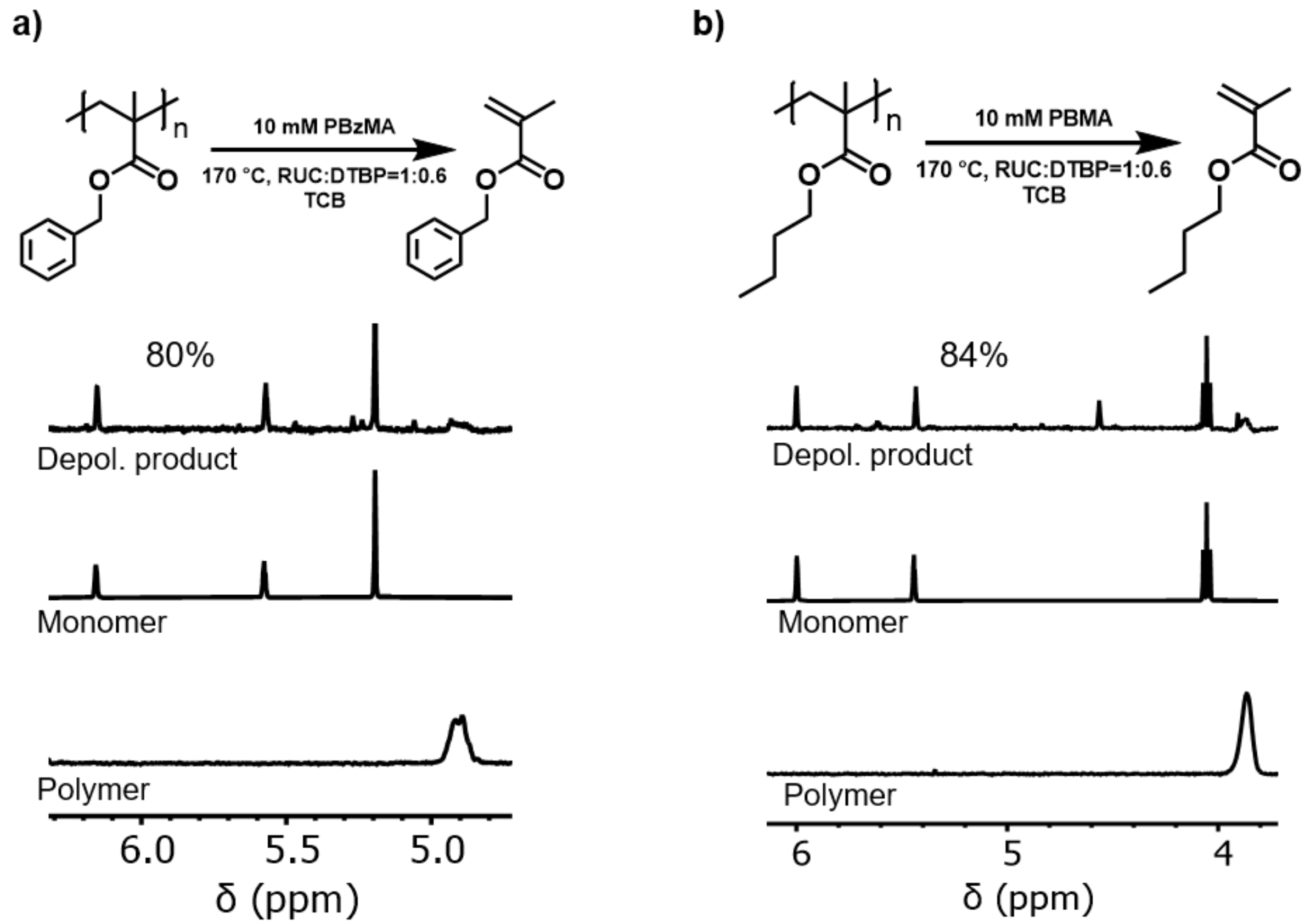

Figure S14 a) Depolymerization of poly(benzyl methacrylate) using DTBP in TCB, and b) depolymerization of poly(butyl methacrylate) using DTBP in TCB, determined via ^{1}H NMR analysis.

References

(1) He, M.; Huang, X.; Zeng, Z.; Yang, J. Phototriggered base proliferation: a highly efficient domino reaction for creating functionally photo-screened materials. Macromolecules 2013, 46 (16), 6402–6407.

(2) Kruželák, J.; Sýkora, R.; Hudec, I. Vulcanization of rubber compounds with peroxide curing systems. Rubber chemistry and technology 2017, 90 (1), 60–88.

(3) Garwood, J. J.; Chen, A. D.; Nagib, D. A. Radical polarity. Journal of the American Chemical Society 2024, 146 (41), 28034–28059.

(4) Luo, Y.-R. Comprehensive handbook of chemical bond energies; CRC press, 2007.
